# Strong context dependence in the relative importance of climate and habitat on nation‐wide macro‐moth community changes

**DOI:** 10.1111/1365-2656.70107

**Published:** 2025-08-04

**Authors:** Emy Guilbault, Pasi Sihvonen, Anna Suuronen, Ida‐Maria Huikkonen, Juha Pöyry, Anna‐Liisa Laine, Tomas Roslin, Marjo Saastamoinen, Jarno Vanhatalo

**Affiliations:** ^1^ Research Centre for Ecological Change, Organismal and Evolutionary Biology Research Programme, Faculty of Biological and Environmental Sciences University of Helsinki Helsinki Finland; ^2^ Finnish Museum of Natural History University of Helsinki Helsinki Finland; ^3^ Finnish Environment Institute (SYKE), Nature Solutions Unit Helsinki Finland; ^4^ Department of Evolutionary Biology and Environmental Studies University of Zürich Zürich Switzerland; ^5^ Spatial Foodweb Ecology Group, Department of Agricultural Sciences University of Helsinki Helsinki Finland; ^6^ Spatial Foodweb Ecology Group, Department of Ecology Swedish University of Agricultural Sciences Uppsala Sweden; ^7^ Helsinki Institute of Life Science University of Helsinki Helsinki Finland; ^8^ Department of Mathematics and Statistics, Faculty of Science University of Helsinki Helsinki Finland

**Keywords:** biodiversity, environmental change, hierarchical modelling of species communities, Lepidoptera, spatio‐temporal change, variance partitioning

## Abstract

1. Habitat modification and climate change are major threats to biodiversity. Understanding the magnitude of their impact and their relative contributions across large spatial scales is important but challenging, given potential context dependence and biases arising from data gaps. Here, we apply a novel predictive framework to tackle these challenges in a study of community data across multiple climatic zones.
2. We used joint species distribution modelling to analyse moth species' occurrence and abundance variation in response to the climate and habitat characteristics within a landscape of 109 sites across Finland spanning 23 years. To correct for spatiotemporal gaps in survey data, we used predictions from the fitted models to evaluate the relative importance of individual drivers on species' occurrence and abundance variation, as well as their effects on community diversity, across the entire country (i.e. beyond the sampled sites). To characterise potential context dependence, we extended model predictions with conditional variance partitioning analysis across species grouped by their functional similarity (functional context) and across sites grouped by the most dominant habitat types (environmental context). Finally, to assess the magnitude of each driver's effect alone on community‐level change, we implemented separate scenario predictions for changes in climate only vs. changes in habitat only.
3. Our results show that the dynamics of communities depended on both functional and environmental contexts. Across Finland, variation in species occurrence was mostly explained by habitat characteristics, but the relative importance of climate and habitat varied according to species functional characteristics and to the dominant habitat types within a landscape. Consequently, temporal trends in community diversity varied across space, and temporal predictions based on changes in climate only vs. changes in habitat characteristics only missed important features of the realised community dynamics.
4. Our results underpin the importance of habitat composition as a key driver of community change—even among temperature‐sensitive ectotherms. Climate and habitat contributed unequally to species occurrence and abundance, and consequently, predictions based on a single driver of environmental change misrepresented realised community dynamics. To understand and predict community change, we thus need to account for the imprints of both climate and land use.

## Graphical abstract

We present a novel predictive variance partitioning and scenario framework for drivers of species and communities. By addressing data gaps and incorporating climatic conditions, habitat, and species traits, our approach reveals the importance of taxonomic and environmental context in shaping responses to environmental change across large spatial scales.

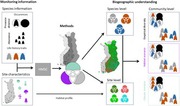

## Tiivistelmä

1. Elinympäristöjen muokkaaminen ja ilmastonmuutos ovat merkittäviä uhkia biodiversiteetille. Niiden vaikutuksen suuruuden ja suhteellisen merkityksen ymmärtäminen laajoilla maantieteellisillä alueilla on tärkeää mutta haastavaa, koska muutosten vaikutukset ovat kontekstisidonnaisia ja tutkimusaineistojen alueellisessa ja ajallisessa kattavuudessa on vaihtelua. Tässä tutkimuksessa sovellamme uutta ennustavaa lähestymistapaa näiden haasteiden ratkaisemiseksi analysoidaksemme yhteisöaineistoja useilta ilmastovyöhykkeiltä.
2. Käytimme lajilevinneisyysmallinnusta ja 23 vuoden aikasarjaa 109:ltä näytteenottoalueelta analysoidaksemme ilmasto‐ ja elinympäristötekijöiden vaikutusta yöperhosten esiintymisen ja runsauden vaihteluun. Korjasimme aineistossa olevia alueellisia ja ajallisia aukkoja sovitettujen mallien ennusteilla arvioidaksemme yksittäisten tekijöiden merkitystä lajien esiintymisen ja runsauden vaihtelulle sekä niiden vaikutusta yhteisön monimuotoisuuteen koko maan laajuisesti (eli myös näytteenottopaikkojen ulkopuolella). Mahdollisen kontekstisidonnaisuuden tunnistamiseksi laajensimme mallien ennusteita ehdollisen varianssihajotelma‐analyysin avulla. Ryhmittelimme lajit niiden toiminnallisen samankaltaisuuden mukaan (toiminnallinen konteksti) ja tutkimuspaikat hallitsevien elinympäristötyyppien perusteella (ympäristökonteksti). Arvioidaksemme yksittäisten tekijöiden vaikutuksen suuruutta yhteisötason muutoksiin, toteutimme myös erilliset skenaarioennusteet ilmastonmuutokselle ja elinympäristön muutokselle.
3. Tuloksemme osoittavat, että yhteisöjen dynamiikka riippui sekä toiminnallisesta että ympäristöllisestä kontekstista. Suomen laajuisesti lajien esiintymisen vaihtelua selittivät pääasiassa elinympäristötekijät, mutta ilmaston ja elinympäristön suhteellinen merkitys vaihteli lajien toiminnallisten ominaisuuksien sekä maiseman hallitsevien elinympäristötyyppien mukaan. Tämän seurauksena yhteisöjen monimuotoisuuden ajalliset muutostrendit vaihtelivat alueittain, ja ennusteet, jotka perustuivat ainoastaan ilmastonmuutokseen tai elinympäristötekijöiden muutoksiin, eivät täysin kyenneet kuvaamaan toteutunutta yhteisödynamiikkaa.
4. Tuloksemme korostavat elinympäristön koostumuksen merkitystä yhteisömuutosten keskeisenä ajurina – jopa lämpötilaherkillä vaihtolämpöisillä lajeilla. Ilmasto ja elinympäristö vaikuttivat lajien esiintymiseen ja runsauteen eri tavoin, minkä seurauksena ennusteet, jotka perustuivat vain yhteen ympäristömuutoksen tekijään, eivät kuvastaneet toteutunutta yhteisödynamiikkaa. Yhteisömuutosten ymmärtämiseksi ja ennustamiseksi on siksi tärkeää huomioida sekä ilmaston että maankäytön vaikutukset.

## 1 INTRODUCTION

Biodiversity changes are ubiquitous, with multiple anthropogenic drivers underpinning those changes (Bowler et al., 2020; Burns et al., 2016). Habitat loss and degradation (Jaureguiberry et al., 2022; Pereira et al., 2012), together with climate change (Mazor et al., 2018), are widely recognised as key drivers of ongoing biodiversity changes. Assessing the relative effects of these drivers is important to mitigate future biodiversity loss (McEntire et al., 2021)—but not trivial in practice (Côté et al., 2016; Santos et al., 2021). Changes in habitat and climate realise differently across space and time. Moreover, species' sensitivity to habitat and climatic changes is shaped by their traits (Hällfors et al., 2023; Hill et al., 2021; Mimet et al., 2019; Schippers et al., 2021). Thus, variation in species sensitivity across space and time can result in reshuffling of species and communities across multiple taxa and at different scales in myriads of ways (Antão et al., 2022).

Studies on the relative effects of climate and habitat on species communities have to date either focused on few species (Clement et al., 2019; Inoue et al., 2019), small spatial or temporal scales (Uhl, Wölfling, & Fiedler, 2022) or failed to account for important spatiotemporal dependencies between the environmental drivers (e.g. climate and landscape scale habitat availability) (Howard et al., 2015) or functional (e.g. morphological and ecological traits) dependencies between the species (Howard et al., 2020; Moudrý & Šímová, 2013). In this study, we simultaneously addressed *environmental* (dominant habitat types within landscape herein) and *functional* (dietary preference and body size herein) context dependency in the relative importance of habitat and climate on biodiversity change.

Within an environmental context, site‐level variation in habitat types may differ between climate zones, and vice versa, implying that the relative importances of environmental drivers on species occurrence and abundance may change between them (Howard et al., 2020; Moudrý & Šímová, 2013). Effects of climate and habitat on species may also be specific to spatial scale and habitat type (Riva et al., 2023; Slade et al., 2013), or constrained by the habitat connectivity within the surrounding landscape (Sonntag & Fourcade, 2022). Within a functional context, the relative importance of climate and habitat characteristics may differ between species depending on their ability to adapt to change (Hof, 2021; Urban et al., 2016). The relative importance of climate and habitat may also differ depending on species characteristics, such as life history strategy, diet, habitat specificity, and dispersal ability (Amburgey et al., 2021; Antão et al., 2022; Howard et al., 2020; Wagner et al., 2021). Therefore, community‐level re‐organisations may depend on the environmental context mediated by the species (i.e. the functional context) present in a community (Montràs‐Janer et al., 2024; Van Klink et al., 2020).

Model‐based variance partitioning has emerged as a tool to summarise and interpret regression‐like models to answer the question of how variability in environmental drivers translates into variability in species occurrence or abundance (Peres‐Neto et al., 2006; Schulz et al., 2020; Viana et al., 2022). Just recently, it was extended to a predictive approach that allows studies on context dependency in these processes (Schulz et al., 2025). Predictive variance partitioning also provides approaches to extend the analyses to larger spatiotemporal domains that are not thoroughly represented by the sampling data (Schulz et al., 2020, 2025). In this work, we take advantage of these recent advances, which have not been applied to community data before, to study the relative importance of climate and habitat on macro‐moth community changes across multiple bioclimatic zones and decades.

Moths are an abundant and species‐rich group of insects and provide a particularly interesting group for addressing context dependence in the drivers of community change. Insects have decreased in diversity, especially in more industrialised parts of Europe (Blumgart et al., 2022; Conrad et al., 2006; Seibold et al., 2019; Valtonen et al., 2017; Van Klink et al., 2020). Yet, the declines are not ubiquitous (Crossley et al., 2021; Pilotto et al., 2020), suggesting different mechanisms of response in different places. Within Finland, species diversity changes in moths follow a latitudinal gradient (Antão et al., 2020), which can be linked to species' strategies to adapt to climate change (range and phenology shifts) and species' responses within their climatic niches (Antão et al., 2022; Hällfors et al., 2021, 2023; Pöyry et al., 2011). Yet, little is known about the relative importance or potential interplay between climate and habitat changes in driving changes in moth communities (Yazdanian et al., 2023).

To investigate the relative roles of climate and habitat as drivers of biodiversity change and to assess their functional and environmental context dependency, we capitalised on some of the longest and highest‐quality time series of moth community composition. These time series span 23 years, 109 unique sampling sites, and almost 60,000 unique sampling occasions across Finland (Huikkonen et al., 2024). A priori, we hypothesised that both climatic and habitat variability will drive species distributions and abundances (Hypothesis 1) but that the relative importance of these drivers will be specific to the environmental (habitat type) and functional context (dietary preference and body size) in the communities (Hypothesis 2). We also expect that this variability in species‐specific responses to climate and habitat leads to patterns in species diversity that are not predictable by climate or habitat only (Hypothesis 3).

To verify these expectations, we first combined data on Finnish moth communities with fine‐scale information on climate and habitat. By extending the Hierarchical Modelling of Species Communities framework (Ovaskainen & Abrego, 2020) into a predictive variance partitioning framework, we then analysed how species occurrence and abundance patterns vary along climatic and habitat conditions (Hypothesis 1), and whether these responses are environmentally or functionally context‐dependent (Hypothesis 2). Drawing on the insights gained at the level of species responses, we then predicted temporal changes of moths at the community level (i.e. species richness and evenness) at a fine scale across Finland—thereby relating community variability to changes in climatic conditions and habitat characteristics (Hypothesis 3).

## 2 METHODS

### 2.1 Moth monitoring data and study sites

To characterise community structure and spatiotemporal change among moths, we used occurrence and abundance observations from the Finnish moth monitoring scheme (Nocturna). This scheme has been running across Finland since 1993 (Huikkonen et al., 2024). Our data comprise 109 sites sampled every night from early spring to late autumn using ‘Jalas’ light traps (Jalas, 1960, supplementary C). For each individual trap, the length of the moth monitoring season has been constant across years. However, between traps, the exact length of the trapping season varies, reflecting regional differences in climatic conditions which allow for a longer trapping period in southern areas. Taxonomically skilled volunteers have emptied all traps weekly and counted the numbers of individuals per moth species (see Appendix S1A) arriving at count data on moth abundances. We formed occurrence data for our hurdle model (see Section 3.1) by deriving binary presence absence of species from the abundance observations, Our analyses were focused on years 1998–2020, which is the period for which both habitat and climate data were available and targeted the moths' main flight period (1 April to 15 October). Though rare species can give valuable information on specialised habitat use, the low sample numbers of such species might interfere with the statistical power of the methods for analyses. Therefore, we only considered macro‐moth species with a prevalence higher than 10%. This resulted in a total of 56,966 species occurrences (26.8% of all occurrences) and a total abundance of about 3.5 million individuals (68% of the total abundance) for 1196 trap‐years (i.e. cumulative occurrences over a season at a trap site) across 78 species (out of 1431 observed species in Finland during our study period).

### 2.2 Moth traits

We used traits to explain moth occurrence and abundance responses to environmental change (see Section 3.1) and to analyse the functional context dependency of the relative importance of climate and habitat (Hypothesis 2). To this end, we considered two features of each species: the wing span of females (W; measured from tip‐to‐tip in mm) and their larval host plant use. These features were chosen to obtain a proxy for species dispersal ability and resource specialisation (Slade et al., 2013; Valtonen et al., 2017; Wölfling et al., 2020), respectively. While wing span is a continuous variable and used as such in Section 3.1, for context dependence analyses (*Context dependency of importance of environmental drivers* (Hypothesis 2)) we grouped moth species by wing span into six groups to match the categorisation level of the other functional trait studied: *W* = 0 mm (*n* = 4), 17.9 < *W* < 24.1 mm (*n* = 16), 24.1 mm < *W* < 30.3 mm (*n* = 16), 30.3 mm < *W* < 36.5 mm (*n* = 30), 36.5 mm < *W* < 43 mm (*n* = 11), and *W* = 55.5 mm (*n* = 1) (Table S1.1; Figures S1.1 and S1.5C,D). Moths were also categorised into six groups according to their principal larval host plant use (either exclusively or mostly based on expert opinion (Yazdanian et al., 2023; Table S1.1; Figure 3a,b)): species with an affinity for (1) herbaceous plants, (2) graminoid plants, (3) dwarf shrubs, (4) deciduous trees, (5) conifers and (6) other food sources (including, e.g. lichens, algae, litter and mushroom). All information on wing span and host plant use follows the terminology from Yazdanian et al. (2023) and Hällfors et al. (2021).

### 2.3 Habitat characteristics

To measure the importance of habitat on moth communities, we characterised the habitat around the traps based on information from the CORINE land cover (CLC) database (Feranec, 2016), as available for years 2000, 2006, 2012 and 2018. We converted CLC data to a pixel resolution of 20 × 20 m using the R package *terra* (Hijmans, 2021) and classified each pixel into habitat categories (for the relation to original CORINE classes, see Table S1.2). Then, building on the general understanding of habitat effects on moths (Habel et al., 2019; Merckx et al., 2019; Öckinger et al., 2010; Appendix S1B), we calculated the following habitat characteristic features over 500 × 500 m^2^ buffers around sampling sites:

1. Proportions of the four habitat categories (broad‐leaved forest, coniferous forest, mixed forest, and semi‐natural or herbaceous habitat).
2. Fragmentation of all forest patches estimated with a clumpiness index using the r package *landscapemetrics* (Hesselbarth et al., 2019; Wang et al., 2014).
3. Diversity of habitat types estimated with the marginal entropy index from the *landscapemetrics* r package (Hesselbarth et al., 2019).

The buffer size was chosen to provide a representation of the surrounding landscapes at a scale relevant for moths (Blumgart et al., 2022). Nonetheless, the buffer sizes used in the literature vary widely among studies (Pascual et al., 2022; Slade et al., 2013). Thus, the scale chosen here (25 ha) offered a compromise between the landscape characteristics and the utility space of the focal organisms.

### 2.4 Climatic covariates

To measure the importance of climate on moth communities, we focused on four climate features that have been shown to be important for moths in previous studies (Neuvonen & Virtanen, 2015; Pöyry et al., 2011, 2018; Uhl, Wölfling, & Bässler, 2022; Appendix S1B): summer growing degree‐days (i.e. thermal sum), winter chilling degree‐days, winter time snow depth, and spring and summer time precipitation. All climatic covariates were derived from 10 km × 10 km gridded data provided by the Finnish Meteorological Institute (FMI; Aalto et al., 2016). Growing degree‐days were calculated as the cumulative sum of temperatures over 5°C during the sampling season (from 1st of April till mid‐October). The cumulative summer time precipitation was calculated for the same period of time, as based on daily precipitation values. In calculating winter chilling degree‐days, we followed Delgado et al. (2020) in summing negative deviations from 5°C (as the reference temperature) during the winter preceding the sampling (from 15 October till 1 of April). Average winter time snow depth for the winter preceding sampling was extracted from daily measurements of snow depth.

### 2.5 Prediction grid

As characteristic for the majority of biodiversity data, the moth survey sites provide a relatively sparse and non‐representative sample of Finnish habitat characteristics and climatic conditions with large spatial gaps in the data (Figures S1.3, S2.4 and S2.5). Similarly, temporal gaps between years with uneven sampling of sites are also present (Figure S1.4d). Hence, to provide results over the whole of Finland, and over the whole study period, we predicted moth occurrence and abundance over a lattice grid of 3 × 3 km resolution covering the entire country for each year between 1998 and 2020 (see Section 3.1). We then analysed the relative contributions of climate and habitat characteristics to changes in moth species occurrence and abundance (Hypothesis 1), the context dependency on them (Hypothesis 2), and the resulting predictability on community diversity (Hypothesis 3) across the grid (see Section 2.6). Climate and habitat covariate values were extracted for each grid node as detailed above.

### 2.6 Statistical analyses

#### 2.6.1 Joint species distribution modelling

To analyse and predict the effects of climate and habitat characteristics on moth occurrence and community composition, we fitted a hierarchical joint species distribution model to aggregated annual moth data (sum of counts over all yearly samples) using the R package Hmsc (Tikhonov et al., 2020). Hmsc fits multivariate Bayesian generalised linear mixed models to community data, thus allowing the joint modelling of data from multiple species and community‐level features (such as species traits or phylogeny—of which only the former is used here) while accounting for spatiotemporal structures of the survey design (Ovaskainen et al., 2017). To make use of information on both species occurrence and abundance, we implemented a hurdle model, fitting two separate parts: a presence–absence (probit) model and an abundance model conditional on presence (i.e. a log‐normal model restricted to observations of positive abundances (in section 3.5.5 of Ovaskainen and Abrego (2020))).

Yearly species information was matched to climatic information calculated at the year level (Section 2.4) and to habitat information that was year‐centred (CLC year 2000 was used for species year‐traps 1998 to 2003, etc.). To adjust for variation in sampling effort (see Section 2.1), we included the log of the number of days for which the traps were operated as a linear term in the model. Moreover, we accounted for the study design by including random effects for site, year and four boreal bioclimatic regions: north boreal, central boreal, south boreal and hemiboreal (Ahti et al., 1968). We also included trait information (see Section 2.2) in the prior of the fixed effects (see section 6.3 in Ovaskainen & Abrego, 2020).

To allow for non‐linear environmental responses, we introduced quadratic polynomial terms for all covariates except for the fragmentation and landscape diversity measures. Fragmentation was modelled with a linear term only, since the existence of an ‘optimal’ fragmentation level seems biologically unrealistic (Hanski, 2015). For the terms including second‐order polynomial responses, Hmsc does not allow constraints on the response shape. Thus, it would be technically possible to observe ecologically unrealistic U‐shaped responses. To identify possibly unrealistic responses, we analysed all the individual environmental response curves (Appendix S1H). These analyses showed that over 99% of all 624 responses in both model components were ecologically realistic, even though we had not restricted the response curves in our model.

#### 2.6.2 Establishing the role of climate vs. habitat in determining species and community distributions

To study the relative importance of environmental drivers across non‐uniformly sampled spatial domains, and their context dependence, Schulz et al. (2025) proposed two extensions to the basic form of model‐based variance partitioning through a scenario‐based predictive approach. We implemented this dual approach as follows.

##### Relative importance of environmental drivers (Hypothesis 1)

To assess the relative importance of environmental drivers on moth occurrence and abundance, we used Hmsc model to predict species occurrence and abundance over the prediction grid covering the whole population of interest (here, Finland over years 1998–2020) after which we calculated a variance partition over this grid. This predictive variance partitioning approach is needed to quantify the general importance of the climatic and habitat covariates across Finland for all years, since the moth sampling locations are a non‐representative sample of all Finnish habitat characteristics and climatic conditions (see Section 2.5).

##### Context dependency of the importance of environmental drivers (Hypothesis 2)

To assess the environmental and the functional context dependence in the importance of climatic and habitat variables, we calculated conditional variance partitions over ecologically interesting subsets of grid sites (environmental context) and species (functional context). To examine environmental context dependence, we clustered the grid sites into four groups of different dominant habitat types within the landscape around the prediction sites classified by k‐means clustering on habitat proportions (Appendix S1C). These individual profiles showed clear differences in their habitat characteristics and were correspondingly labelled as forest (dominated by forest), water (dominated by water), semi‐natural (dominated by wetland, vegetation and forest) and heterogeneous profiles (Figure S1.3). To examine functional context dependence, we grouped the species according to their wing span and host plant use as detailed in Section 2.2.

##### Role of climate and habitat in determining community composition and variability (Hypothesis 3)

The analyses above were targeted to estimate the importance of climatic vs. habitat influences on individual species. At the community level, differential imprints on individual species are likely to translate into changes in community *composition*. As the community‐level outcome of species‐level effects, we explored changes in the richness and Hill–Simpson evenness (Heip et al., 1998) of communities over space and time.

We first calculated overall species richness and evenness from the presence–absence and the abundance predictions, respectively, over the full prediction grid over Finland for each year. To further analyse how climate and habitat change might each have affected community structure over the past two decades, we made two counter‐factual scenario predictions of community evenness and richness across Finland over the study years. In the *habitat scenario*, the values of habitat covariates corresponded to their site‐specific, empirically measured values, but the values of climatic covariates were fixed to the average of their values during the first 5 years of the time series. This prediction, thus, mimics the case where only habitat had changed, whereas climate had not—thereby pinpointing the community‐level impacts of habitat change alone. In the *climate scenario*, the values of climatic covariates varied from year to year following their site‐specific, empirically measured values, but the habitat covariates were fixed to their values in year 2000. This prediction, thus, pinpoints the community‐level impacts of climate change alone. For each scenario, we calculated the trend and variability of community‐level diversity measures by fitting independent linear regression models, with year as a covariate. The local trends were then measured by the estimates of the regression coefficients, and the local variability was measured by the variance of the standardised residuals of the linear models.

## 3 RESULTS

### 3.1 Variation in moth occurrences and abundances

**FIGURE 1:**
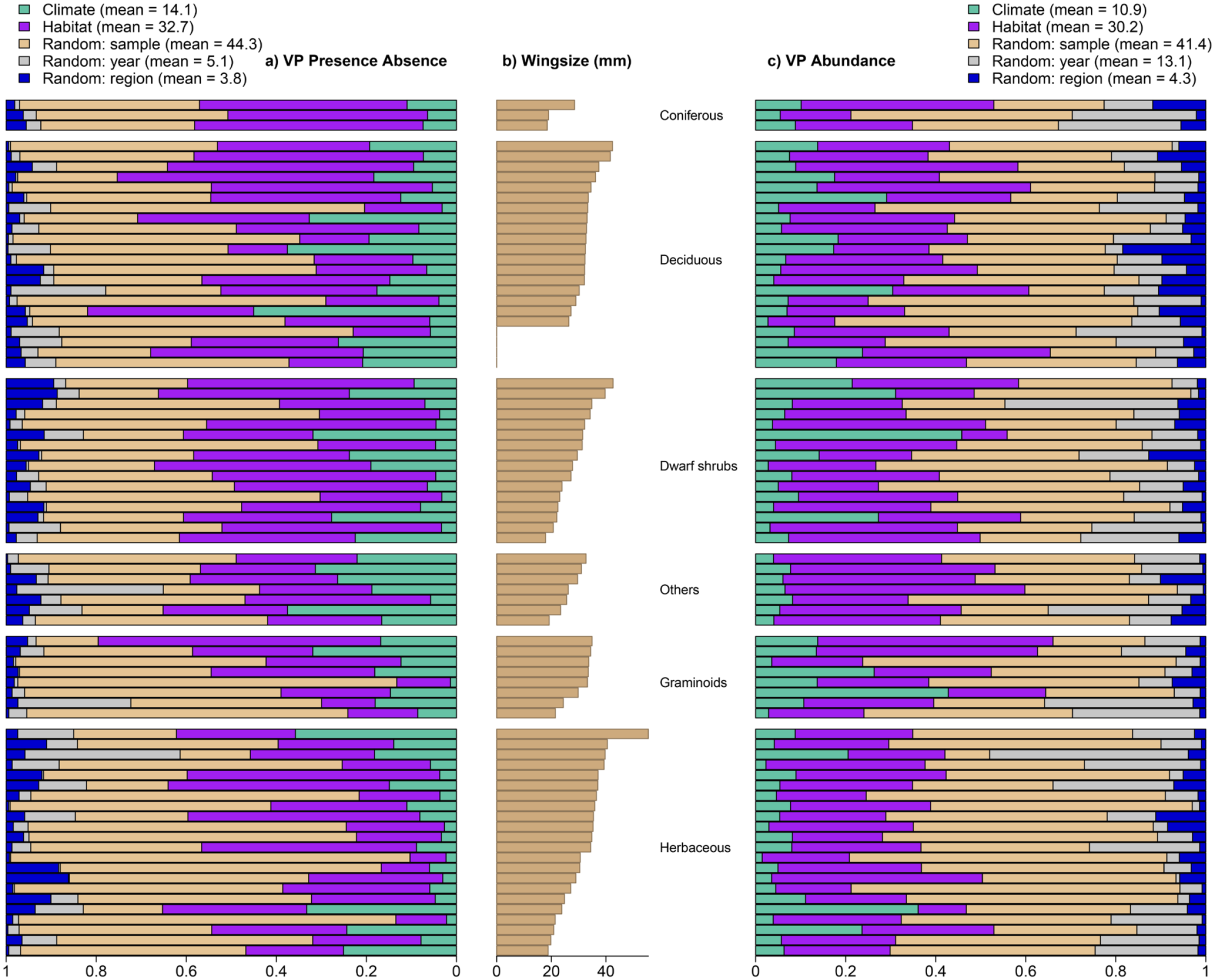
The proportion of variation (VP) in species occurrence (a) and abundance conditioned on presence (c) explained by environmental covariates as fixed effects and spatial and temporal covariates as random effects across the predictive sites (Finland). Each row represents a species, and species are grouped by host plant growth form (as shown between b and c). Within each hostplant group, individual species are sorted by increasing wing span (from bottom to top—b). The rows order matches the order in Table S1.1.

The explanatory and predictive powers were good in both the presence–absence (explanatory Tjur*R* ^2^ 0.50 and AUC 0.92; predictive Tjur*R* ^2^ = 0.27 and AUC = 0.81) and the abundance component of the hurdle model (*R* ^2^ 0.58; 0.13) (Table S2.1). Covariates associated with habitat and climate together explained approximately half of the variation in moth occurrences of the 78 focal species over Finland, contributing respectively 33% (with 95% CI of [15; 55]%) and 14% ([4.1; 29]%) of the total variance (Figure 1). The other half of the variation in species occurrence was explained by random effects at the levels of site (44% [26; 64]%), year (5.1% [1.9; 11]%) and bioclimatic zone (3.8% [0.1; 9.7]%). Out of the 78 focal species, habitat was the dominant driver of occurrence for 63 ([56; 70]) species and of abundance for 67 ([62; 72]) species.

In terms of variation in moth abundances, conditional on presence, covariates associated with habitat and climate explained 30% ([12.4; 51.9]%) and 11% ([3.2; 24.3]%) of the variance, respectively (Figure 1). The site‐level random effect explained 41% ([25; 60]%), while year and bioclimatic zone random effects explained 13% and 4.3% of the variation in species abundance conditional on presence (with 95% CIs of [4.7; 28]% and [0; 14]%).

**FIGURE 2:**
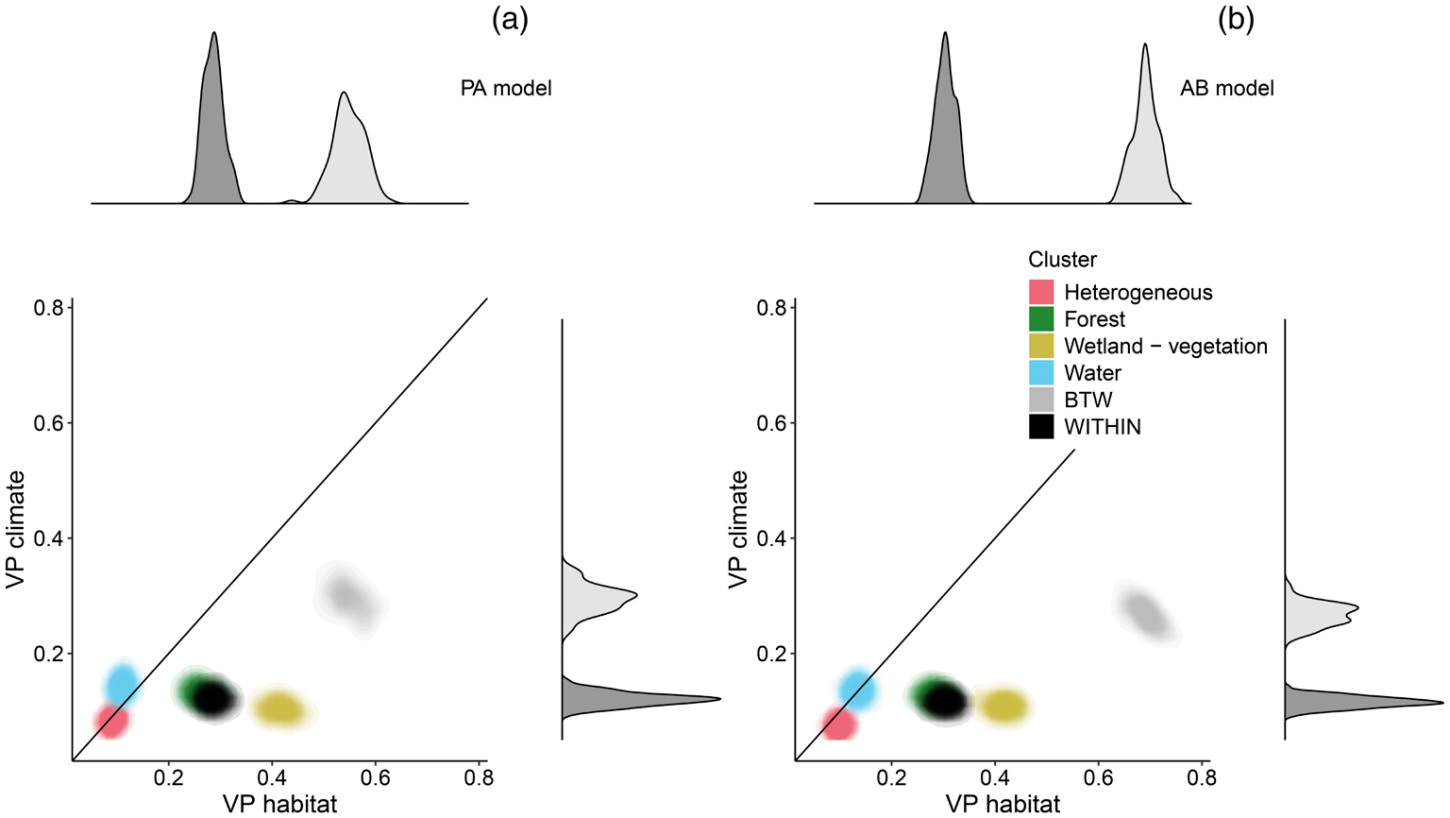
Context dependence of the importance of habitat vs. climate for moth occurrence (PA (presence/absence); subplot a) and abundance (AB; subplot b) over Finland. The heat plots in panels (a) and (b) show the joint posterior distributions of the proportion of variance explained (VP) by habitat (*x*‐axis) and climate (*y*‐axis) across locations within each of the four main habitat profiles (heterogeneous, forest, wetland, and water; red to blue colours) and the partition of the total variance into variation WITHIN and between (BTW) habitat profiles (black and grey colours). The marginal posterior distributions of the within‐between partition are shown by the density plots along the margins of the figures.

In the environmental context dependence analysis, the importance of climate for moth occurrences and abundances was almost constant among all four clusters of dominant habitat type (Figure 2). The importance of habitat, on the other hand, varied considerably between these clusters so that the proportion of variation explained by habitat covariates was largest for forest and wetland profiles and smallest for water and heterogeneous profiles.

**FIGURE 3:**
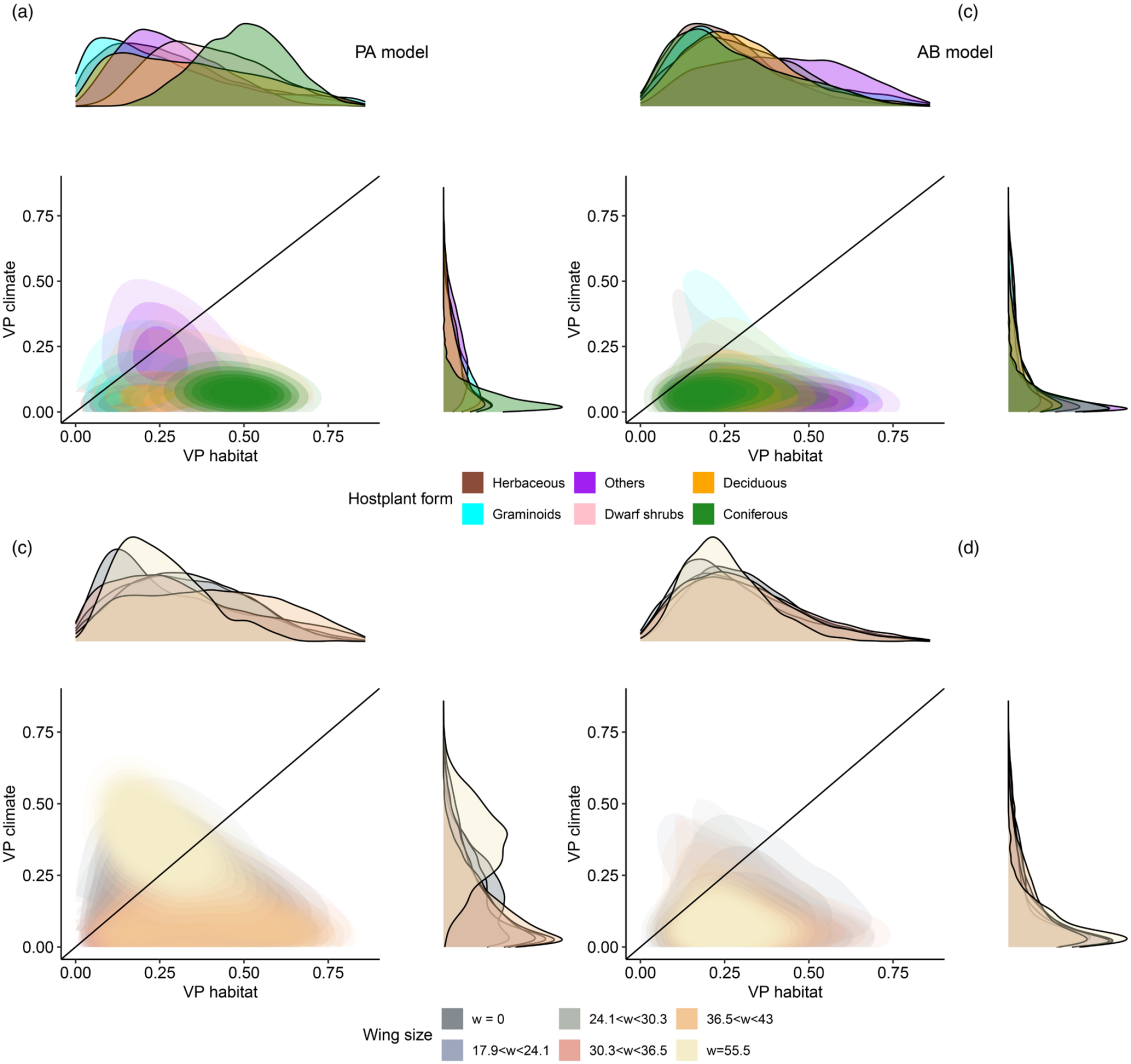
Variation in the importance (VP) of habitat vs. climate for the occurrence (PA (presence/absence) model; panels a and c) and abundance (AB model; panels b and d) of moths associated with different traits over Finland. The top row (panels a and b) shows results for species grouped by host plant growth form, whereas the bottom row (panels c and d) shows results for species grouped by wing span. Each subplotsummarisess the joint posterior distributions (heatmap) of the proportion of variance explained by habitat (*x*‐axis) vs. climate (*y*‐axis) among species sharing the same growth form of their host plants (graminoids, herbaceous plants, dwarf shrubs, coniferous trees, deciduous trees and other food sources) or sharing similar wing span (*W* = 0 mm, 17.9 < *W* < 24.1 mm, 24.1 mm < *W* < 30.3 mm, 30.3 mm < *W* < 36.5 mm, 36.5 mm < *W* < 43 mm and *W* = 55.5 mm).

In the analysis of functional context dependence, the importance of climate and habitat for moth occurrence varied substantially between species with different types of host plant use (Figure 3a). The occurrence of moths associated with coniferous and deciduous host plants varied relatively more than the occurrence of the other species along habitat characteristics, whereas climate showed relatively higher explanatory power for moths in the host plant group other food sources. However, for species abundance conditional on presence, we observed no context dependence in the importance of climate and habitat among moth host plant groups (Figure 3b). When species were categorised based on their wing span, the relative contributions of climate and habitat were similar across all other species except *Noctua pronuba*, which has by far the largest wing span (*W* = 55.5 mm) among the studied species, and the four species with wingless females (*Agriopis aurantiaria*, *Erannis defoliaria*, *Operophtera brumata*, *Operophtera fagata*) (Figure S1.1). For these five species, climate explained a relatively larger proportion of variance than for the other species (Figure 3c,d).

### 3.2 Variation in the diversity of moth communities

**FIGURE 4:**
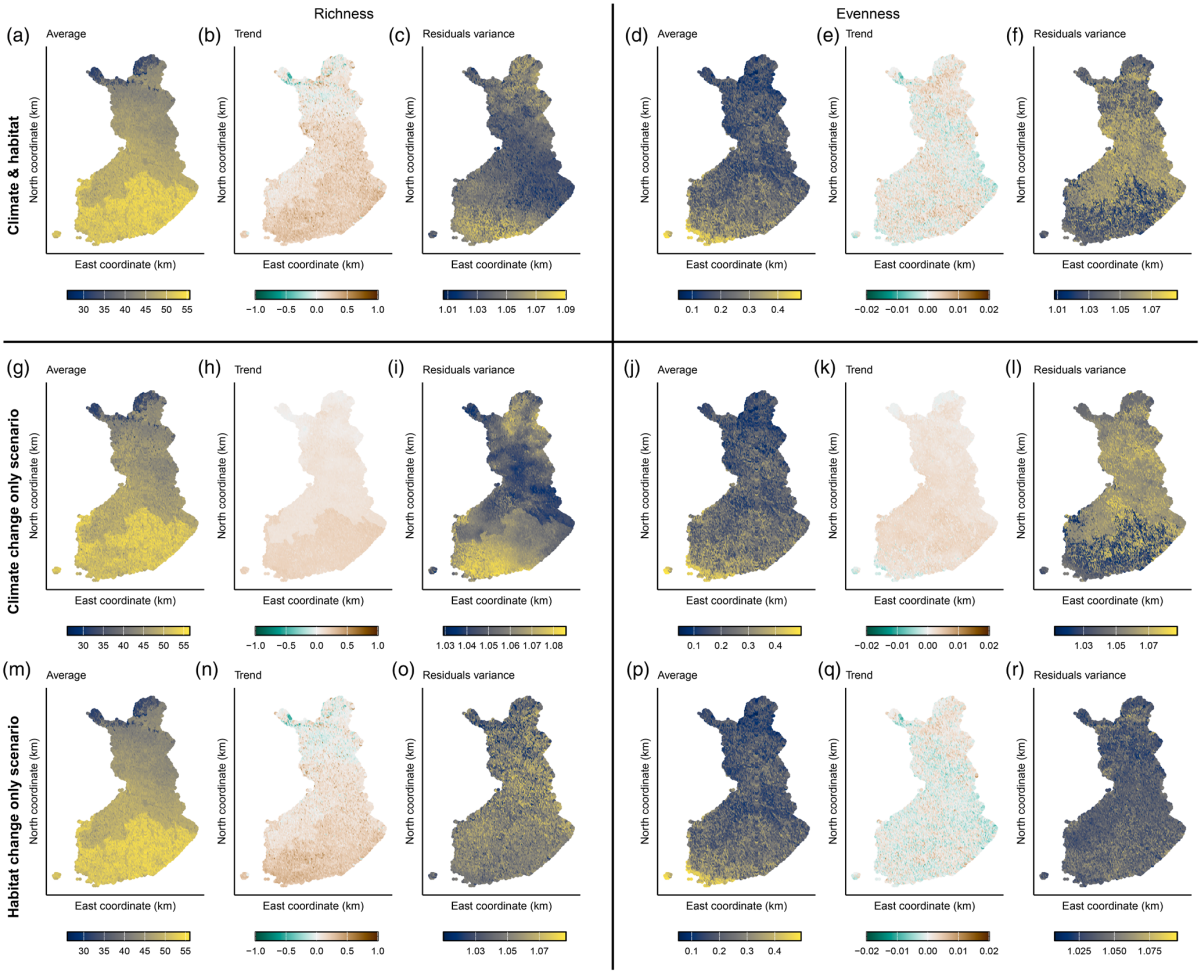
Summary of predicted diversity across years in Finland. On the left, we display the predicted species richness of moth communities across the national grid of sites, and on the right, we show predicted species evenness. Panels on row 1 show the summary of predictions between 1998 and 2020 with both climate and habitat variables, in terms of the average across the period (a and d), the local (point‐wise) trend estimated from a linear model (b and e), and the variability in standardised residuals (variance; c and f). Panels on rows 2 and 3 from the top show summaries of predictions for the two scenarios of climate change only (g–l) or habitat change only (m–r).

The myriad of ways individual moth species responded to climate and habitat covariates was reflected in spatial and temporal patterns in moth communities' richness and evenness (Hill‐inverse Simpson's evenness) as well as in changes in these metrics. Both species richness and evenness showed a latitudinal gradient, which was more pronounced in the patterns of species richness (Figure 4a,d). Richness also increased over years consistently across Finland, with a strengthening trend towards the south, whereas trends in evenness varied from increase to decrease in a heterogeneous manner across Finland. The yearly variability of species richness and evenness around the temporal trends was higher in the north than in the south (Figure 4c,f).

In the scenario predictions with climate change only (keeping the habitat fixed; Figure 4g–l), the long‐term mean and yearly variation in species richness and evenness matched well the predictions including both climate and habitat (Figure 4a–f). Opposite to this, the scenario predictions involving changes in habitat only (keeping the climate fixed; Figure 4m–r) were similar to the predictions with both climate and habitat in terms of the average over years and temporal trends, but not in terms of the temporal variation. The climate‐only vs. habitat‐only scenarios differed notably in their temporal trends.

## 4 DISCUSSION

Biodiversity is undergoing unprecedented changes due to ongoing climate change and habitat alterations—but the relative importance of these two drivers for changes in natural communities remains poorly understood, with only a few earlier studies on birds (Clement et al., 2019; Eglington & Pearce‐Higgins, 2012; Howard et al., 2015, 2020; Moudrý & Šímová, 2013), mammals (Rubidge et al., 2011), bees (Kammerer et al., 2021), butterflies (Kivinen et al., 2007) and moths (Uhl, Wölfling, & Fiedler, 2022). In this study, we were specifically interested in understanding the relative importance of climate and habitat in defining species occupancy, abundance and diversity. The observed patterns in occurrence and abundance shed light on different facets of biodiversity distribution and change, thus demonstrating that environmental drivers and their relative importance can differ in explaining range variation (occurrences) and population size or community composition and structure (abundance) (Howard et al., 2014; Schulz et al., 2020). As a key novelty, we explicitly studied the environmental and functional context dependence of the importance of climate and habitat drivers on moth communities.

### 4.1 Habitat is a major driver of moth communities

Even though the importance of climate and habitat in defining local species' and communities' distribution patterns are widely studied, few studies have assessed their relative importance for any taxa. Most of these studies have highlighted the dominance of climatic drivers (Clement et al., 2019; Howard et al., 2015; Kivinen et al., 2007) (Kammerer et al., 2021; Rubidge et al., 2011), while habitat has rarely been identified as the dominant driver (Eglington & Pearce‐Higgins, 2012; Kadlec et al., 2009; Uhl, Wölfling, & Fiedler, 2022). In our results, variability in both moth occurrence and abundance is best explained by various habitat characteristics (e.g. the proportion of forest cover and of lower vegetation, as well as patch configuration). Even though *both* climatic and habitat factors were important in explaining the observed variability in the moth community encompassing 78 focal species in line with Uhl, Wölfling, and Fiedler (2022), habitat was the dominating explanatory factor of occurrence (abundance) for 63 (67) species. Similarly, the scenario predictions of moth communities suggest that spatiotemporal trends in Finnish moth community compositions are mainly driven by habitat, whereas climate drives temporal community variability. The differences between our results and the earlier results may be explained by differences between focal taxa and study region, but also by the spatial scale at which habitat characteristics and climate information have been encoded into the analyses (Riva et al., 2023; Slade et al., 2013). Moreover, earlier results for Finnish moths have revealed a latitudinal variability of the climate inducing increases in species richness and decreasing abundance over time (Antão et al., 2020, 2022). Yet, the total abundance and biomass of moths have remained largely unchanged when explained by climatic conditions and forest proportion alone (Yazdanian et al., 2023).

### 4.2 Environmental and functional context dependence in the relative importance of climate vs. habitat

We demonstrated that the importance of climatic conditions and habitat characteristics on species occurrence and abundance is not uniform across the country or among communities. Instead, it depends on the environmental and functional context. This finding aligns with previous results on both micro and macro moths (Uhl, Wölfling, & Fiedler, 2022), birds (Howard et al., 2015, 2020; Moudrý & Šímová, 2013) and bees (Kammerer et al., 2021). In terms of environmental context, the imprints of habitat vs. climate varied across sites with different dominant habitat types. Moth occurrences in more vegetated sites, that is, those dominated by forest and by semi‐natural habitat, were driven relatively more by variability of habitat. This may reflect higher diversity in forest landscapes in terms of levels of fragmentation (Wölfling et al., 2019, 2020) and management practices (Merckx et al., 2012). In contrast, habitat and climate were roughly equally important for moth communities in heterogeneous and water‐dominated sites. These sites are primarily composed of disturbed (i.e. urban and agricultural habitats) or open habitats, which are likely more vulnerable to climate variability (De Frenne et al., 2021). Our current results add credence to the notion that certain habitats may be better at buffering the impacts of climatic variability than others (De Frenne et al., 2021; Outhwaite et al., 2022; Suggitt et al., 2011).

In a functional context, we found that the resource use (i.e. host plant type) of species influenced the relative importance of climate and habitat in driving species occurrence, with lesser impact on their relative importance to species abundance. Among the focal species, tree feeding species expressed the highest variability along habitat characteristics, whereas climate had a relatively larger effect on moths in the host plant group other food sources. Wing span, on the other hand, had little impact on relative importance of habitat or climate on species occurrence or abundance—even though this trait is often used as a proxy of dispersal ability or resource specialisation (Slade et al., 2013; Valtonen et al., 2017; Wölfling et al., 2020; Yazdanian et al., 2023). Only the species with a clearly larger wing span than that of the rest of the studied species, and the species with wingless females, stood out with climate being a relatively more important driver of occurrence than for the other species. The imprints of host plant use—and the mostly undetectable imprint of wing span—underscore the challenge of linking coarse trait information and coarse habitat definition, to patterns of changes in species occurrences (cf. Blumgart et al., 2022; Hällfors et al., 2021), especially when shaped by multiple drivers. Even though the habitat characteristics provided a more accurate division of host plant availability for tree feeding species than for herbaceous species (see also Section 4.5), the observed importance of diet preferences appears intuitive, as access to host plants will directly affect species' habitat use, rendering habitat specialists more sensitive to habitat characteristics than to climate change (Kadlec et al., 2009; Uhl, Wölfling, & Fiedler, 2022; Wagner et al., 2021).

### 4.3 Community diversity under environmental changes

Our results on community‐level species richness and evenness patterns agree with earlier findings of an increase in Finnish macro‐moth richness over time and a decrease in average species richness and evenness with latitude (Antão et al., 2020, 2022). The patterns of temporal trends and residual variation in species richness and evenness (Figure 4) can partly be explained by high species turnover in boreal latitudes (Ellis et al., 2024; Mäkinen et al., 2025), which can reflect into community homogenisation (Valtonen et al., 2017; Yazdanian et al., 2023). For moths, such diversity dynamics may be driven by spatiotemporal variation in food availability and quality (Blumgart et al., 2022; Pöyry et al., 2017)—with added imprints of other traits, such as thermal preferences (or combinations of multiple traits) (Yazdanian et al., 2023).

Predictions based on separate changes in climate or habitat allowed us to study the imprints of these drivers on community structure. Thus, we could isolate and dissect the imprints of individual drivers on community change—thereby highlighting potential mitigation or synergy in space and time. Taken together, our results from these scenario predictions suggest that spatiotemporal changes in Finnish moth communities are mainly driven by land use disturbances, while climate impacts are mainly evident for temporal fluctuations. In addition, we show that the northern regions in Finland express the highest variability in communities' diversity. Nonetheless, habitat changes have greatly contributed to the decrease in species richness in the north. Over the country, they generated less homogeneous communities. Overall, our results emphasise that temporal predictions based solely on changes in a single driver—whether climate or habitat characteristics—overlooked key aspects of the realised community dynamics.

### 4.4 Correcting for biases arising from data gaps is important

On a more methodological note, our study demonstrates the importance of the predictive variance partitioning approach (and modelling in general) for population level analysis when the sampling design is unrepresentative of the total area, or of the time period, under study (Bowler et al., 2025; Foster et al., 2021; Schulz et al., 2025). Given the large latitudinal range of Finland (1100 km in a north–south direction), different parts of the country show widely different climatic and habitat conditions. Importantly, the habitat and climate characteristics at the 109 moth survey sites did not adequately represent those across whole Finland and whole study time (see Appendix S1B and S1C). While the actual sampling sites suffice to reveal imprints of climate and habitat (Bowler et al., 2025), as such they represented a biased sample of all habitat and climate conditions over Finland, and of temporal changes in these. As a result, the estimates on relative importance of climate and habitat on moth communities would have been biased if they had been calculated across the sampled sites only (Foster et al., 2021; Schulz et al., 2025; for comparison between analyses over sampling sites only vs. across whole Finland, see Appendix S2). Non‐representative sampling designs are common in long‐term ecological data. However, the majority of the existing studies where the interest concerns a large non‐uniformly sampled study area, and time interval, apply variance partitioning to sampling sites while ignoring the bias in population level estimates induced by the sampling design (but see Yuan et al. (2017) and Schulz et al. (2020)). Our predictive variance partitioning approach is applicable to community analyses well beyond this work to overcome both spatial and temporal gaps. To enhance its adoption in other systems, we provide all our code in generalisable form in Appendix S3.

### 4.5 Sensitivity of the results to model assumptions

As all statistical analyses of observational data, our results depend on a number of assumptions. First, because of methodological constraints in analysing rare species in Hmsc, our results represent only the most abundant moth species (see Section 2.1). If the rarer moth species responded to climate and habitat differently from the common species considered here, the conclusions on their relative importance over the whole moth community could change. However, our results still represent most of the moth biomass in Finland since the studied species represented 68% of the total number of observed specimens in the moth monitoring data over a 23‐year period. Second, a finer grained division of functional properties of moths could lead to a more comprehensive understanding of the context dependency of the relative importance of climate and habitat among them. However, larval host plant type and wing span correlate with important differences in life cycle strategies among moths (Mangels et al., 2017; Slade et al., 2013; Valtonen et al., 2017; Wölfling et al., 2020; Yazdanian et al., 2023) and those traits were available for all moth species. Moreover, while the larval host plant type is naturally categorical, we had to artificially discretise the wing span to carry out the conditional variance partitioning. The results were not sensitive to this discretisation as long as the species with the highest wing span and the species with wingless females were put into their own categories. Third, even though our covariates reflect the ecology of the studied species, both the climatic data and the CORINE Land Cover data are low‐resolution descriptions of the environment. Hence, they miss information on microclimate and plant species richness, composition and diversity, which could allow for constructing a more direct link between habitat and moth communities at different spatial scales (Coelho et al., 2023; Uhl, Wölfling, & Fiedler, 2022) and to pinpoint more precisely climatic buffering effects (De Frenne et al., 2021).

## 5 CONCLUSIONS

Overall, our study suggests that habitat contributes more than climate to variability in the occurrences and abundances of Finnish moths. However, the relative importance of these two types of drivers is highly context‐dependent, varying among species sharing similar wing span and larval host plant use traits and especially among sites with different dominant habitat types. Our study further provides a template for understanding and quantifying the context dependence of habitat and climatic drivers on community assembly, and for examining their relative imprints, from non‐homogenously sampled data (i.e. data with spatiotemporal gaps). As such, it also reveals the dangers involved in inferring specific trends from opportunistically designed monitoring schemes. To overcome these limitations, we show how we may use predictions to bridge the sample to the actual population of inference—here the population of moth communities in Finland. When combined, these insights provide important steps towards understanding complex patterns of community change in space and in time.

## AUTHOR CONTRIBUTIONS

E.G., M.S., J.V., T.R. and A.‐L.L. conceived the ideas and designed the methodology; P.S., A.S., I‐M.H. and J.P. collected the data; E.G. analysed the data; E.G. and J.V. led the writing of the manuscript. All authors contributed critically to the drafts and gave final approval for publication.

## CONFLICT OF INTEREST STATEMENT

The authors do not have any conflict of interest to report.

## ETHICAL APPROVAL

The work includes research on invertebrates, which do not need ethical permissions. Details on the fieldwork are presented in Huikkonen et al. (2024). Permission from the landowner is obtained for the maintenance of the trap, and in cases when the trap is located in a protected area, a permission for trapping is obtained from the nature conservation officials (Metsähallitus or ELY centre).

## STATEMENT ON INCLUSION

Local data collection was led by the Finnish Environment Institute (SYKE) for species occurrences, and the Finnish Museum of Natural History for species information. The Finnish moth monitoring scheme (Nocturna) is a long‐term monitoring programme that relies on local volunteer data collection across the country.

## Supporting information


**Appendix S1.** Supplementary methods.
**Appendix S2.** Supplementary results.
**Appendix S3.** Code and data.

## Data Availability

Data available from the Dryad Digital Repository https://doi.org/10.5061/dryad.0k6djhb5r (Guilbault et al., 2025).
