# Supplementary material for: Strong context dependence in the relative importance of climate and habitat on nation‐wide macro‐moth community changes
Source: J Anim Ecol. 2025 Aug 4;94(10):1948–61. doi: 10.1111/1365-2656.70107 (PMC12484425; doi:10.1111/1365-2656.70107)
Supplement: Supplementary file 1 — Appendix S1. Supplementary methods. Appendix S2. Supplementary results. Appendix S3. Code and data. [file JANE-94-1948-s001.docx]

Supplementary material

Strong context-dependence in the relative effects of climate and habitat on macro-moth community changes in Finland

Contents

[Appendix S1: Supplementary methods 3](#_Toc195887200)

[A- Moth identification information 3](#_Toc195887201)

[Table S1.1: Moth species and trait information. 3](#_Toc195887202)

[Figure S1.1: Moth species wing span distribution. 4](#_Toc195887203)

[B- Justification for land-use and climate variables 5](#_Toc195887204)

[Figure S1.2: Environmental drivers correlation matrix. 6](#_Toc195887205)

[C- Habitat classification using clustering 6](#_Toc195887206)

[Table S1.2: Habitat categories characterising the sampling sites and grid sites within Finland. 6](#_Toc195887207)

[Figure S1.3: Characteristics of the four habitat profiles at grid sites across Finland (left) and at moth sampling sites (right). 7](#_Toc195887208)

[D- Summary statistics on sites and observations 7](#_Toc195887209)

[Figure S1.4: Summary of Moth observations and sites sampled. 8](#_Toc195887210)

[Figure S1.5: Numbers of observations per habitat profiles and trait categories. 9](#_Toc195887211)

[E- HMSC modelling 9](#_Toc195887212)

[F- HMSC Variance partitioning 10](#_Toc195887213)

[G- Is there a time lag in species response? A model comparison 11](#_Toc195887214)

[Table S1.3: WAIC comparison of the hurdle models with alternative temporal delays in habitat covariate. 12](#_Toc195887215)

[H- What type of species response curve do we observe? 12](#_Toc195887216)

[Figure S1.6: Classification of response curve types when using second order polynomial response functions. 13](#_Toc195887217)

[Table S1.4: Summary statistics on the ‘undefined’ response curve shapes over the covariate range over the sampling sites. 13](#_Toc195887218)

[Table S1.5: Summary statistics on the ‘undefined’ response curve shapes over the covariate range over the prediction grid covering whole Finland. 14](#_Toc195887219)

[Figure S1.7: Species response curves with ‘ushape’ like pattern at the sampling sites (A) and at the Finnish grid sites (B). 15](#_Toc195887220)

[Appendix S2: Supplementary results 16](#_Toc195887221)

[A- Model performances 16](#_Toc195887222)

[Table S2.1: Model fit statistics for the hurdle models with habitat delays 16](#_Toc195887223)

[B- Variance partitioning of moth occurrence and abundance – Sampling sites 16](#_Toc195887224)

[Figure S2.1: Variance partitioning among grouped covariates (habitat vs. climate) across the sampling sites. 17](#_Toc195887225)

[Figure S2.2: Context-dependence of the importance of habitat vs. climate for moth occurrence (PA) and abundance (AB) at sampling sites. 17](#_Toc195887226)

[Figure S2.3: Variation in the importance of habitat vs. climate for the occurrence (PA (presence/absence) model – panels a and c) and abundance (AB model –panels c and d) of moths associated with different traits at the sampling sites. 18](#_Toc195887227)

[C- Environmental changes within sampling sites and across Finland 19](#_Toc195887228)

[Figure S2.4: Summary of climatic changes in Finland 20](#_Toc195887229)

[Figure S2.5: Summary of habitat changes in Finland. 21](#_Toc195887230)

[Appendix S3: Code and data 21](#_Toc195887231)

[References 21](#_Toc195887232)

# Appendix S1: Supplementary methods

## Moth identification information

Moth materials have been collected by using the “Jalas” light traps (Jalas, 1960) that are equipped with 160 W mixed light or 125 W mercury (Hg) vapour bulbs to attract the moths. Collected moth samples were preserved with tetrachloroethane (or a mix of tetrachloroethane and chloroform) which is much less volatile than chloroform. The small bottle with the liquid inside a trap container easily lasted for several weeks. Traps have been emptied on average once a week, and maintainers of traps were advised to add tetrachloroethane always when they saw that more is needed

All macro-moth species (with the addition of Hepialidae and Cossidae species) have been identified across all trap sites (for the focal families see page 17 in the 30-yr report of the Finnish moth monitoring scheme:(Huikkonen et al., 2024). The samples were identified by highly skilled voluntary lepidopterists who were members of the Lepidopterological Society of Finland (see page 78 in the 30-yr report). Quality control of identifications has been made by the coordination team across all the years. Classification and taxonomy of moth species has been based on the up-to-date official list of Finnish Lepidoptera that currently can be found at the FinBIF hub (<https://laji.fi/en/taxon/list?target=MX.53695&onlyFinnish=true>).

The main text books used in identifications are the following ones:

- Marttila, O., Saarinen, K., Haahtela, T. & Pajari, M. 1996: Suomen kiitäjät ja kehrääjät. - Kirjayhtymä, Helsinki. 384 p.
- Mikkola, K. & Jalas, I. 1977: Suomen perhoset, Yökköset 1. - Otava, Helsinki. 256 p.
- Mikkola, K. & Jalas, I. 1979: Suomen perhoset, Yökköset 2. - Otava, Helsinki. 304 p.
- Mikkola, K., Jalas, I. & Peltonen, O. 1985: Suomen perhoset, Mittarit 1. - Suomen Perhostutkijain Seura, Tampere. 260 p.
- Mikkola, K., Jalas, I. & Peltonen, O. 1989: Suomen perhoset, Mittarit 2. - Suomen Perhostutkijain Seura & Recallmed, Hanko. 280 p.
- Silvonen, K., Top-Jensen, M. & Fibiger, M. 2014: Suomen päivä- ja yöperhoset -maastokäsikirja. - BugBook Publishing, Østermarie. 820 p.

In addition, European-wide book series such as “Noctuidae Europaeae” and “Geometridae moths of Europe” have been in use. There are also nowadays very good internet sources to help identifications, e.g. the following ones are useful in northern Europe:

- <https://lepiforum.org/>(Fält-Nardmann et al., 2018; Virtanen and Neuvonen, 1999)
- <https://www.perhoset.fi/sps_suomen_perhoset.htm>
- <https://mothdissection.co.uk/>

We display below in table S1.1 information about the 78 species used in the analyses and their associated traits categories for hostplant forms and wingspan (See *Moth traits* in the main manuscript). The distribution of wing spans is presented in Figure S1.1.

### Table S1.1: Moth species and trait information.

*The 78 moth species analysed in this study and their associated traits used in the analyses. The colour coding for the wing span trait corresponds to the grouping of wing span into seven equally long wing span categories as shown in Figure S1.1.*


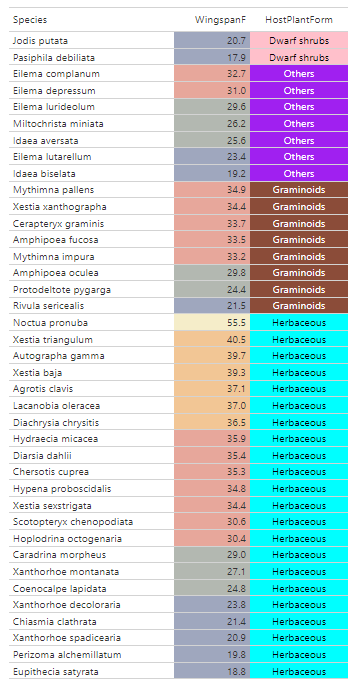

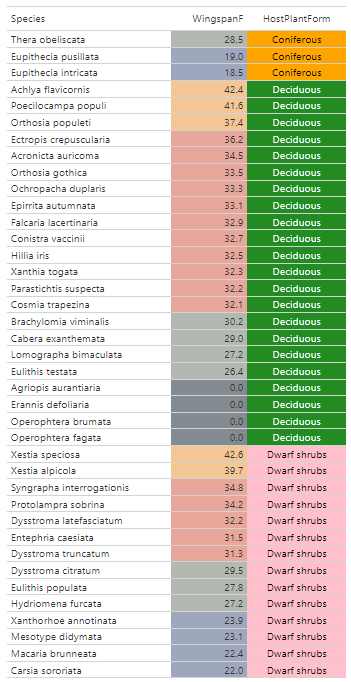


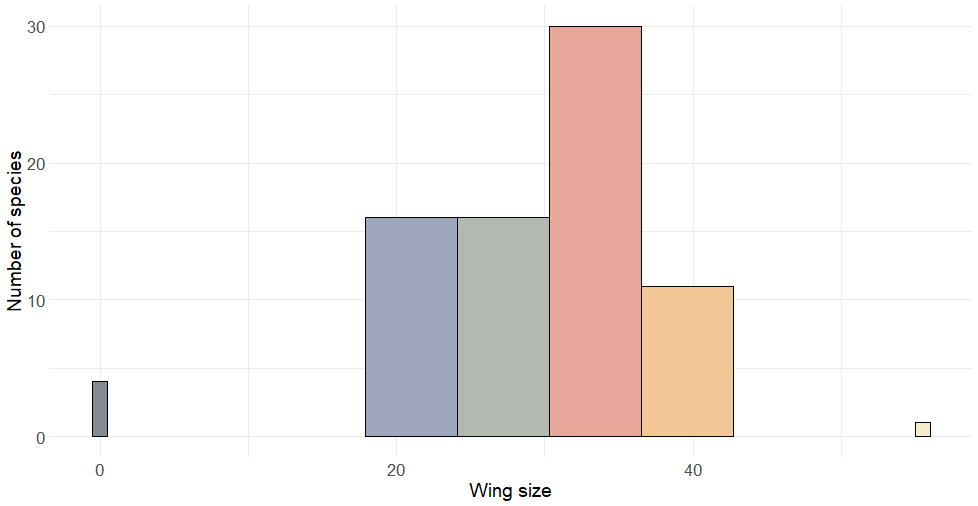


### Figure S1.1: Moth species wing span distribution.

*The histogram shows the number of species per each group of wing spans. W=0mm (n=4), 17.9<W<24.1mm (n=16), 24.1mm<W<30.3mm (n=16), 30.3mm<W<36.5mm (n=30), 36.5mm<W<43mm (n=11), and W=55.5mm (n=1).*

## Justification for land-use and climate variables

To support our model implementation, we based the choice of our variables on studies that used either climatic, habitat or both data to inform moth communities. Previously, the amounts of forest and meadow habitat have been found to positively influence moth species richness (Merckx et al., 2019; Summerville and Crist, 2004), whereas forest fragmentation has been shown to negatively affect moth richness and abundance (Lintott et al., 2014; Öckinger et al., 2010; Slade et al., 2013). In addition, the diversity of habitat types available in the landscape will likely shape the diversity of host plants and microclimatic conditions available, with higher habitat diversity expected to support higher moth richness (Habel et al., 2019; Merckx et al., 2019, 2012).

Regarding climatic variables, the thermal sum has been found to be strong predictors of for both insect phenology (Pöyry et al., 2018), range limits (Luoto et al., 2006), and life-history characteristics (Pöyry et al., 2011). Winter chilling degree-days reflect the frequency and duration of particularly cold temperatures in the winter, which restrict the distribution and survival of (some) moth species (Fält-Nardmann et al., 2018; Virtanen & Neuvonen, 1999). Snow cover and its persistence are key factors when studying northern latitude ecosystems in general (Niittynen and Luoto, 2018). Snow depth can have a dual effect: On the one hand, a deeper snow cover will insulate the ground (where most moth species hibernate) from low winter-time temperatures (Neuvonen and Virtanen, 2015). On the other, much snow in the winter will also contribute to higher spring- and summer-time soil moisture levels (Rissanen et al., 2021) and delay the moth activity period in spring (Pöyry et al., 2018). Finally, spring- and summer-time precipitation, in addition to temperature, are closely related to the availability of soil moisture and habitat quality, thereby affecting both moth hatching success (Esbjerg and Lauritzen, 2010) and moth species richness and community composition (Uhl et al., 2022).

Most environmental drivers show low correlation (below 0.7) (Figure S1.2). The largest correlation and the only one higher than 0.7 was found between snow and the chilling degree days (r = 0.87). Given that we analysed all climate variables together with our grouped variance partition, this did not impact our study results.


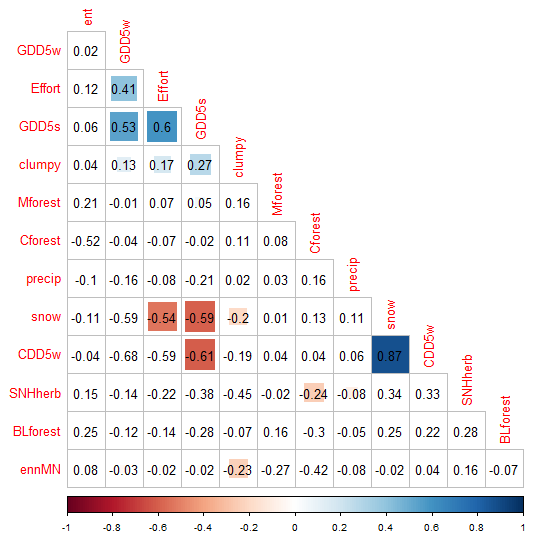


### Figure S1.2: Environmental drivers correlation matrix.

*The correlation matrix displays the correlation between the different environmental drivers used in the models. Positive correlations are coloured in blue while negative correlation in red. The larger the correlation, the larger the square and the darker the colour is.*

## Habitat classification using clustering

To characterise the habitat characteristics of the sampling sites, we used the CORINE Land Cover (CLC) categories (Table S1.2). Here, the summary category ‘semi natural herbaceous’ includes non-forest semi natural areas (shrub and/or herbaceous vegetation, open space with little or no vegetation) and heterogeneous vegetated areas (agriculture land with significant natural vegetation, agroforestry, and pastures).

### Table S1.2: Habitat categories characterising the sampling sites and grid sites within Finland.

*To describe the habitat context of sites, we used CORINE land cover data as pooled into six wider habitat types (column “Habitat type”). Column “CLC categories” identifies the original CORINE classes included in each category. For each habitat we calculated the proportion of pixels classified into the respective category within a 500×500 m buffer area around each of our 109 sampling sites and across all sites resulting from the regular grid being applied over Finland. The relative representation of original CORINE classes in the two materials are summarized in columns “Proportion within study sites” and “Proportion within Finland”.*


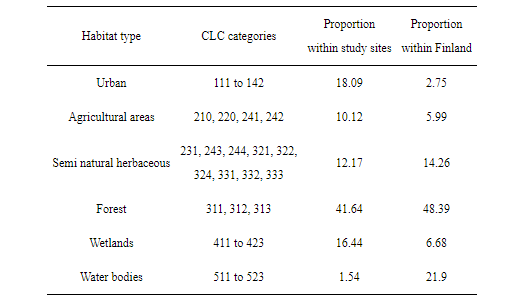


To divide our sites into clusters of dominant habitat based on the above habitat categories proportions, we used the r package clValid (Brock et al., 2021) to compare multiple clustering algorithms ('UPGMA', "hierarchical", 'kmeans', 'diana', 'model', 'sota', 'fanny', 'pam', 'clara', 'agnes') and numbers of clusters (2 to 4) across different distance metrics (‘euclidean’, ‘manhattan’, ‘correlation’). Of the resulting clustering, we chose the consistently best-performing parametrization, as based on seven metrics of performance: connectivity; Silhouette Width; Dunn Index, average proportion of non-overlap (APN); average distance (AD); average distance between means (ADM), and the figure of merit (FOM)). The habitat profiles obtained are presented in Fig. S1.3.

***
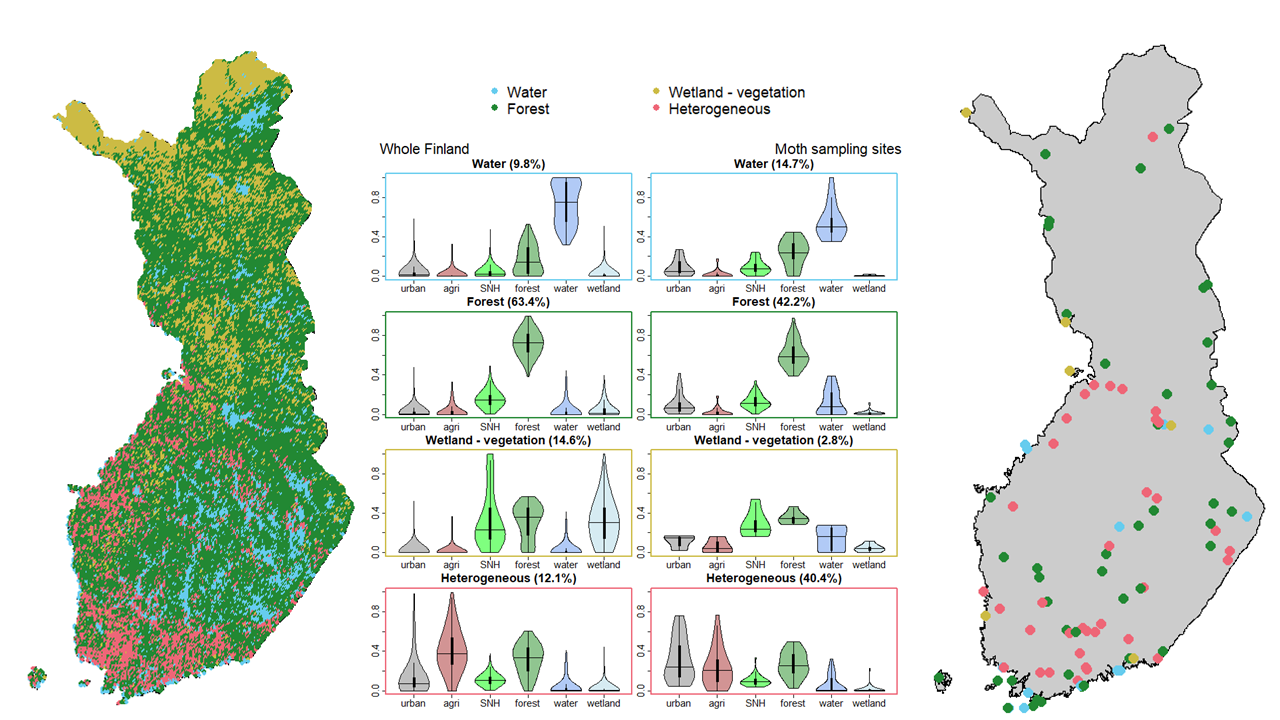
***

### Figure S1.3: Characteristics of the four habitat profiles at grid sites across Finland (left) and at moth sampling sites (right).

*To describe the habitat context of each site, we used a 500x500m buffer square to calculate the proportion of pixels within each habitat category identified in Table S1.1. The violin plots summarise the distributions of habitat proportions within each of the four habitat profiles identified by k-means clustering. The colour of each violin separate habitat groups (urban in grey, agricultural lands in red, semi natural and herbaceous in light green, forest in dark green, water bodies in dark blue and wetland in light blue). The box colour corresponds to the colour of sites of the same habitat profile indicated on the map. Percentages above each panel identify the relative proportion of sites belonging to this habitat profile across Finland (left-hand panels) and across sampling sites (right-hand panels).*

## Summary statistics on sites and observations

The total number of observations (1196 observations across 78 moth species with only species with a prevalence above 10%) offered representative coverage across the main habitat profiles and years (Fig. S1.4) and across different trait categories (Fig. S1.5). Spatio-temporal gaps are discussed in more details in the main manuscript.


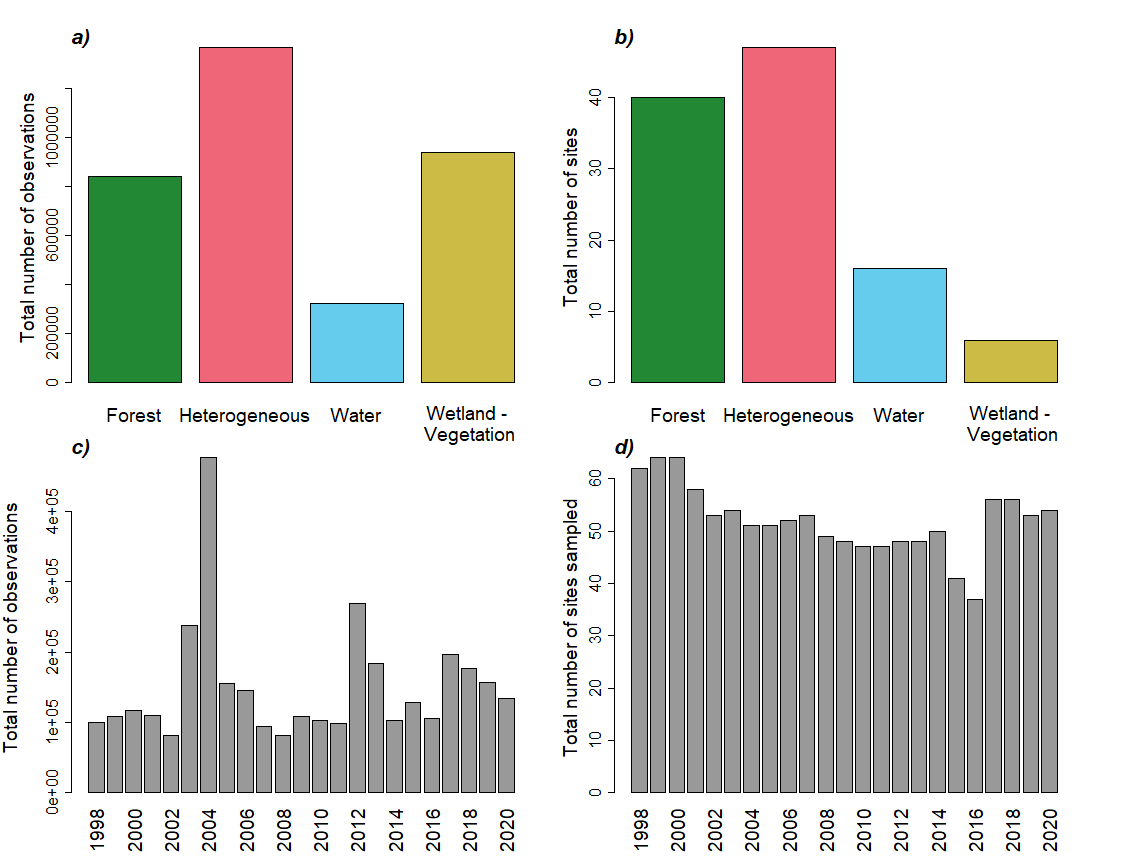


### Figure S1.4: Summary of Moth observations and sites sampled.

*Numbers of observations (A & C) and sites sampled (B & D) across main habitat profiles (A & B) and years (C & D).*

*
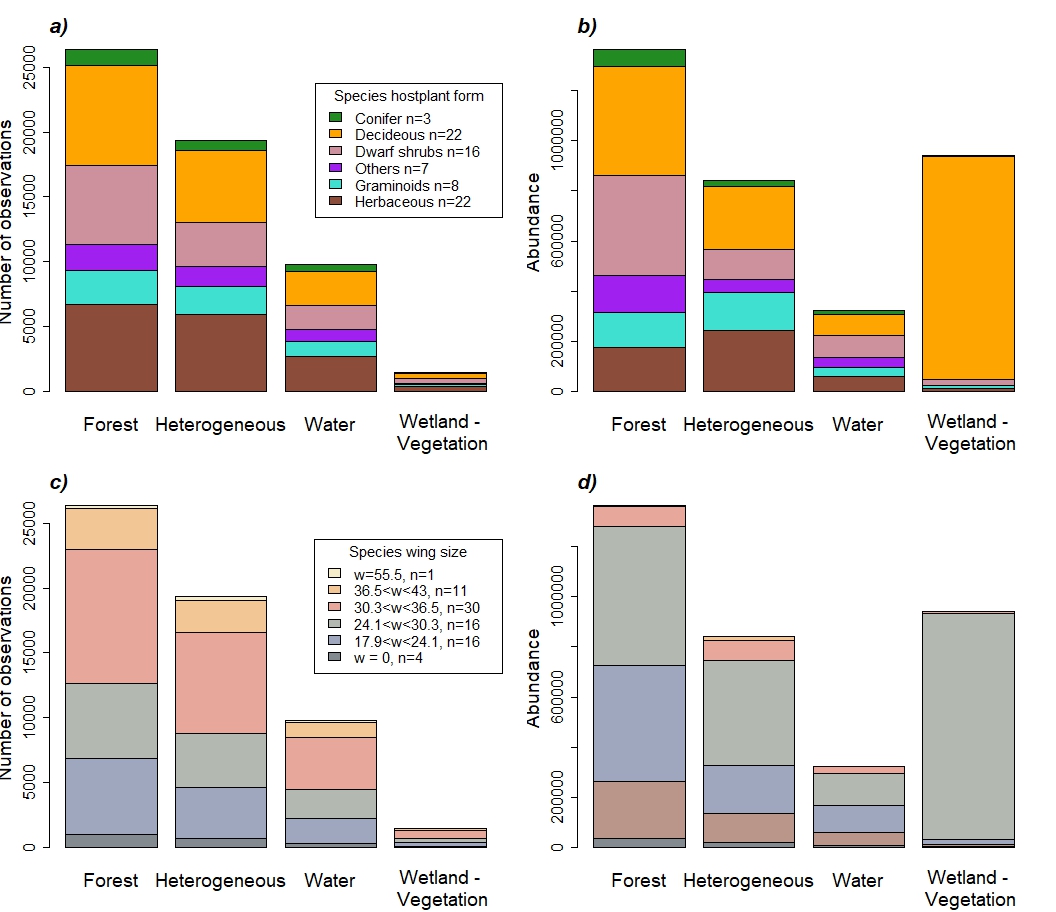
*

### Figure S1.5: Numbers of observations per habitat profiles and trait categories.

*Shown is the total number of moth observations recorded (left) and total abundances (right), grouped by main habitat profile (x-axis). In panels A and B, we group the records by the growth form of the larval host plants; in C and D by wing (W) size (W=0mm, 17.9<W<24.1mm, 24.1mm<W<30.3mm, 30.3mm<W<36.5mm, 36.5mm<W<43mm, and W=55.5mm.). The number of species per trait category is shown in the colour legend.*

## HMSC modelling

We used Markov chain Monte Carlo to sample from the posterior distribution. We run four chains for 375,000 iterations and removed the first 125,000 samples as burn-in. We thinned the chains by 1000 iterations resulting in total of 1000 samples that were used for posterior inference. The MCMC convergence was assessed by visual evaluation of the sample chains and with the potential scale reduction factor (Ovaskainen and Abrego, 2020). Following Ovaskainen & Abrego (2020), we measured the explanatory power of the models by using the area under the curve (AUC) and coefficient of discrimination (Tjur R²) for the presence absence model and the R² for the abundance model. Additionally, we evaluated the predictive power (Tjur R² and R² respectively) by performing a two-fold cross validation on both parts of our model.

## HMSC Variance partitioning

To study the relative importance of environmental covariates and random effects, we thus implemented a series of variance partitioning summary statistics and their posterior uncertainty estimates. In these comparisons, we grouped climatic variables and habitat characteristics, and then compared the relative variance attributable to each group.

The starting point in variance partitioning metrics is the latent Gaussian linear predictor of Hmsc, which can be written as

$$L_{ij}=L_{ij}^{F}+L_{ij}^{R}$$

where $L_{ij}^{F}$ stands for the fixed effects (i.e., the effects of environmental covariates) and $L_{ij}^{R}$ for the random effects (i.e., year, boreal region, and site) of a species j at a sampling occasion i. The basic form of the variance partition of the linear predictor is calculated by partitioning the variance in L_ij over all sampling occasions between (groups of) the fixed (F) and random (R) effects (Ovaskainen et al. (2017). When implemented across the sampling sites, this variance partitioning provides information on the relative importance of different model components at the specific set of sampling locations and times.

Next, to arrive at the population-level variance partition across Finland, we predicted the latent variables at all grid sites covering Finland (Section Prediction grid) and calculated the importance of climatic versus habitat information over them. The conditional variance partition, for context dependence analyses, was implemented by first clustering the grid sites and then classifying the sample sites into four groups of similar habitat characteristics (henceforth referred to as “habitat profiles”) (Supplementary Fig. S1.1 and Supplementary S1A). After this, we calculated the relative contributions of climate and habitat variables, as well as of the random effects, to the variability of the linear predictor within and between these representative habitat profiles (Schulz et al., 2025). To further analyse the context-dependence of the importance of climate and habitat from the point of view of species traits, we also calculated this conditional variance partition separately for subsets of species grouped by their hostplants, or their wing span (categorized to 3 classes).

To extend the variance partitioning implemented in HMSC (Ovaskainen and Abrego, 2020; Tikhonov et al., 2020), we used the framework of Schulz et al. (2025). Formally, we define a grouping function *g(x)*, which assigns each predictive, or training, site into a homogeneity group based on sites habitat profile. The variance partitioning can then be divided into three components:

1. Conditional variance partitioning for each group of habitat profile:

$$V_{k/g=c, j}= \frac{Var(a_{g=c, k, j})}{Var(L_{g=c, j})}$$

Here $a_{g,k,j}$is a fixed linear term over sampling occasions, where *g(x)* assigns observations to a group *c,* for covariate *k* and species *j*.

1. Within-condition variance partitioning within habitat profile groups, which describes the level of similarity in species responses to habitat or climate information within a cluster site type:

$$V_{k}^{wi}= \frac{Var(a_{\cdot,k, j}-a_{\bar{c},\cdot, k,j})}{Var(L_{j})}$$

where $a_{\cdot,k, j}$ is a fixed linear term of the model, and $a_{\bar{c},\cdot,k, j}$ is a per-condition mean of the fixed linear term. This term reaches low values for high levels of within-condition similarity and high values for low levels of within-condition similarity.

1. Between-condition variance partitioning among habitat profiles, which translates to how similar (low values) or distinct (high values) the species responses in one cluster site type is to another:

$$V_{k}^{btw}= \frac{Var(a_{\bar{c},\cdot, k,j})}{Var(L_{j})}$$

This term reaches low values for high levels of between-condition similarity and high values for low levels of between-condition similarity.

To characterise the variance of the random linear predictors, we proceeded analogously. The random linear predictor in HMSC can be decomposed as shown in Ovaskainen et al. (2017):

$$L_{i,j}^{R}= \sum_{l=1}^{L} \varepsilon_{i,l,j}= \sum_{l=1}^{L} \sum_{h=1}^{n_{f}} \eta_{ihl}\lambda_{ljh}$$

where *L* is the number of random effects predictors $\varepsilon$ at a sampling unit *i* for a species *j*, $n_{f}$ is the number of factors, $\eta_{ih}$ are the latent factors, and $\lambda_{jh}$ are the factor loadings.

Thus, we calculated the following variance partitions (Schulz et al., 2025):

1. Conditional variance partitioning:

$$V_{l/g=c, j}= \frac{Var(\varepsilon_{g=c, l, j})}{Var(L_{g=c, j})}$$

where g(x) assigns observations to a group *c, and* $\varepsilon_{g,l,j}$is the random linear term over sampling occasions.

1. Within-conditions variance partitioning:

$$V_{l}^{wi}= \frac{Var(\varepsilon_{\cdot,l, j}-\varepsilon_{\bar{c},\cdot,l,j})}{Var(L_{j})}$$

where $\varepsilon_{\bar{c},\cdot,l, j}$ is the random linear term per-condition mean and $\varepsilon_{\cdot,l, j}$ a random linear term of the model.

1. Between-conditions variance partitioning:

$$V_{l}^{btw}= \frac{Var(\varepsilon_{\bar{c},\cdot, l,j})}{Var(L_{j})}$$

## Is there a time lag in species response? A model comparison

To assess whether current or past habitat explains moth occurrence better, we constructed the habitat covariates with time lags of 10 years, 7 years, 5 years, 3 years and 1 year. For example, for a 5-year time lag; we explained moth observations made in 2004-2009 with the CLC values of 2000 (noting that CLC rasters are available for only every sixth year). To decide on what habitat time lag value to use in the final analyses, we compared the WAIC values of presence -absence and abundance models (see *Community modelling*) (Ovaskainen and Abrego, 2020). We compared the alternative models’ WAIC separately for the occurrence (PA) and abundance conditioned on presence (AB) (Table S1.2). There were no significant differences between the WAIC values of alternative occurrence models (ΔWAIC_max-min_= 0.59). For abundance models, there was no significant difference between the WAIC values of *No delay* and *1 year delay* models (ΔWAIC_1year-NoDelay_= 0.56) whereas the other models were clearly worse than these two models (ΔWAIC_Xyear-NoDelay_ > 3.5) (Table S1.3).

### Table S1.3: WAIC comparison of the hurdle models with alternative temporal delays in habitat covariate.

*Each column represents the delay assumed in matching habitat information to the observation year in the presence-Absence (PA) and Abundance conditioned on presence (AB) model.
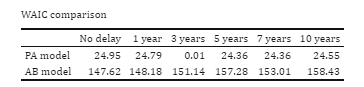
*

## What type of species response curve do we observe?

The Hierarchical Model for Species Communities (HMSC) does not allow any constraints on the species response curves because of computational limitations in the MCMC sampling algorithm underlying its inference machine. Hence, we used unconstrained second order polynomials and retrospectively checked that the inferred responses were ecologically sensible. For example, having a U-shaped species responses on environmental covariates would be technically possible to observe in our model but biologically difficult to interpret.

Before going into the details of our model fit, we first define more clearly what we mean by a U-shaped response. The choice of using first and second order term for a covariate allows us to model various shapes of species responses (Fig. S1.5). Depending on the sign of the weight of the second order term (w) and the derivative of the response curve over the range of observed covariate values (d), the response curve is either linear (w=0), curvilinear (w~=0 and d~=0 within the covariate range), U-shaped (w>0 and d=0 within the covariate range), or bell-shaped (w<0 and d=0 within the covariate range). With a U-shaped response, we mean a model fit which predicts clearly U-shaped response curve along a covariate within the range of observed covariate values. That is, the response curve is first clearly decreasing, has zero derivative, and ends increasing within the range of covariate values. With a bell-shaped response, we mean a model fit which is a negative of U-shaped curve. On the other hand, a curvilinear response curve might have negative or positive second order weight but is monotonic within the range of the covariate values. In case of monotonic responses, the best fit can be achieved with w=0, w<0 or w>0, depending on data. This is the same definition of response types as used by Antão et al. (2022) and is illustrated below.


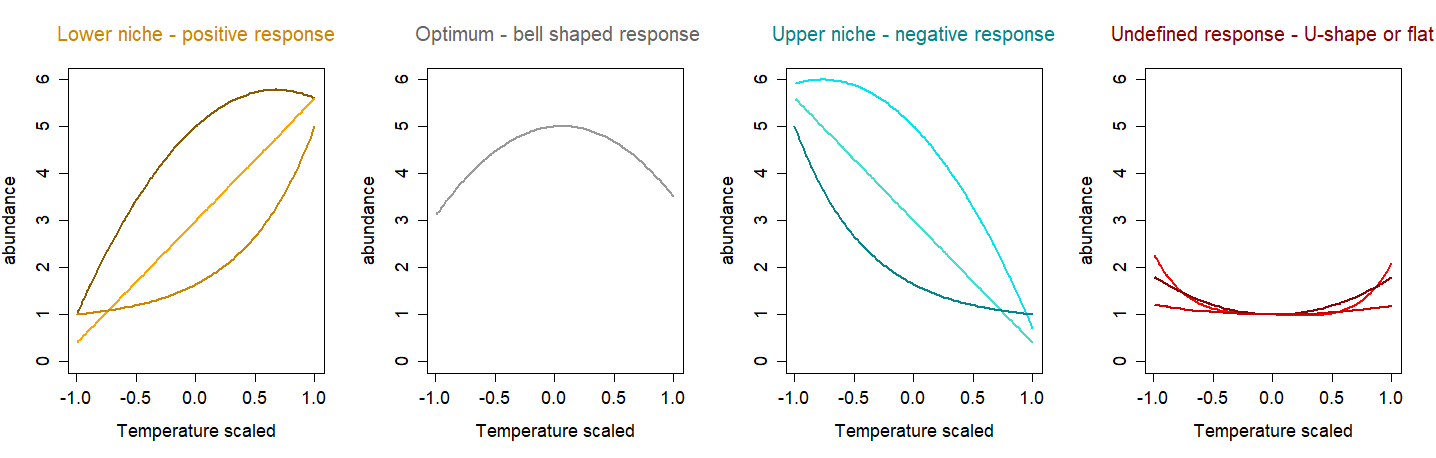


Figure S1.6: Classification of response curve types when using second order polynomial response functions. *The classification is done according to the shape of the response curve within the range of observed covariate values (illustrated by the range of the x-axis in the figure).*

That said, it is still possible to have ecologically sensible convex response curve as long as such a curve is (mostly/in practice) monotonic within the range of observed covariate values – corresponding to curvilinear response (see the first and third subplot in Figure S1.6 above). However, the response can also be truly U-shaped within the covariate values but still ecologically sensible if the variability along the curve is practically zero (see the fourth subplot in Figure 1 above). This is because in these practically “no response” cases the stochasticity of data defines whether the inferred response is (slightly) U- or bell-shaped.

Hence, to study the response curves in our study, we applied the method of Antão et al. (2022) and classified all species and covariate wise responses to increasing, optimum, decreasing, or undefined. The U-shaped responses, if any, would belong to the undefined group. In total, we had 78 species for which 8 covariates were modelled using a second order polynomial which correspond to 624 response curves in total. We calculated the posterior probability for all four types of curves for each of these 624 cases. Because the range of covariates in sampling sites and Finnish grids sites (the prediction grid) are slightly different, we calculated the posterior probabilities for the response types for both cases. Moreover, we calculated the (posterior mean of the) proportion of total variance in the predictor explained by all covariates for each of the species. These variance partitioning (VP) statistics were then used to measure whether the response curves were actually informative for species presence-absence or abundance.

When considering all presence-absence response curves within the covariate range of the sampling locations, only 24 (3.8 %) of them had significant posterior probability (>75 % probability) for undefined response (Table S1.4). However, only 2 (0.32 %) of them were also informative for species presence-absence as measured by explaining more than 10% of the total variance in the predictor. Respectively, in the abundance model, 46 (7.4 %) of responses had significant posterior probability for undefined response but only 1 (0.16 %) of the responses was also informative for species presence-absence. When considering response curves along the covariate ranges over the prediction grid covering whole Finland, the results were practically the same (Table S1.5).

Table S1.4: Summary statistics on the ‘undefined’ response curve shapes over the covariate range over the sampling sites. *Each row corresponds to an interval of posterior probability for having undefined response curve. The columns summarize how many of the individual response curves (and what proportion of them, %) had this posterior probability for undefined response over all response curves (columns 2-3), over response curves that explained 0-5 % of the total variance in the latent factor (columns 4-5), over response curves that explained 5-10 % of the total variance in the latent factor (columns 6-7), and over response curves that explained over 10 % of the total variance in the latent factor (columns 8-9).*

Table S1.5: Summary statistics on the ‘undefined’ response curve shapes over the covariate range over the prediction grid covering whole Finland. *Each row corresponds to an interval of posterior probability for having undefined response curve. The columns summarize how many of the individual response curves (and what proportion of them, %) had this posterior probability for undefined response over all response curves (columns 2-3), over response curves that explained 0-5 % of the total variance in the latent factor (columns 4-5), over response curves that explained 5-10 % of the total variance in the latent factor (columns 6-7), and over response curves that explained over 10 % of the total variance in the latent factor (columns 8-9). sites.*

We next examined visually those 2+1 response curves that had high posterior probability of being undefined (potentially U-shaped), and that were also informative on species presence-absence or abundance, over the covariate range in the sampling sites (Fig. S1.7A). Out of these response curves only one (abundance prediction along coniferous forest proportion for *Protolampra sobrina*) is slightly U-shaped by visual inspection. However, for this response also the curve is mostly increasing.

We examined also those 2+3 response curves that had high posterior probability of being undefined (potentially U-shaped), and that were also informative on species presence-absence or abundance, over the covariate range in the prediction grid (Fig. S1.7B). Out of these response curves, two (probability of presence prediction along mixed forest proportion for *Thera obelisca*, and *Eupitheca intricata togata*) clearly showed a U-shaped response upon visual inspection. This is due to the particular mixed forest proportion gradient at the Finnish grid sites which mostly displayed low mixed forest proportion (<0.6) over most prediction grids (P(Mforest)>0.6 was observed in less than 1% of the prediction grid sites each year). This extreme environmental gradient for Mixed forest did not exist along the sampling sites (max(P(Mforest))=0.41).The three others (abundance prediction along semi natural herbaceous habitat proportion for *Hydriomena furcata*, *Orthosia gothica* and *Xanthia togata*) were slightly U-shaped by visual inspection. Observing these U-shaped response curves could potentially be driven by the combination with other covariates or be the result of complex co-occurrence system that can exist in communities considered in joint species distribution models. In addition, extreme environmental gradients are not uncommon across a large spatial scale and could also trigger more ‘disturbed’ convex species response shapes as well as other factors (Anderson et al., 2022; Jansen and Oksanen, 2013).

Since over 99 % of the response curves over the total of 624, were ecologically sensible (either increasing, decreasing or bell-shaped), we conclude that our results are valid even though we have not restricted the response curves to be concave in our model.


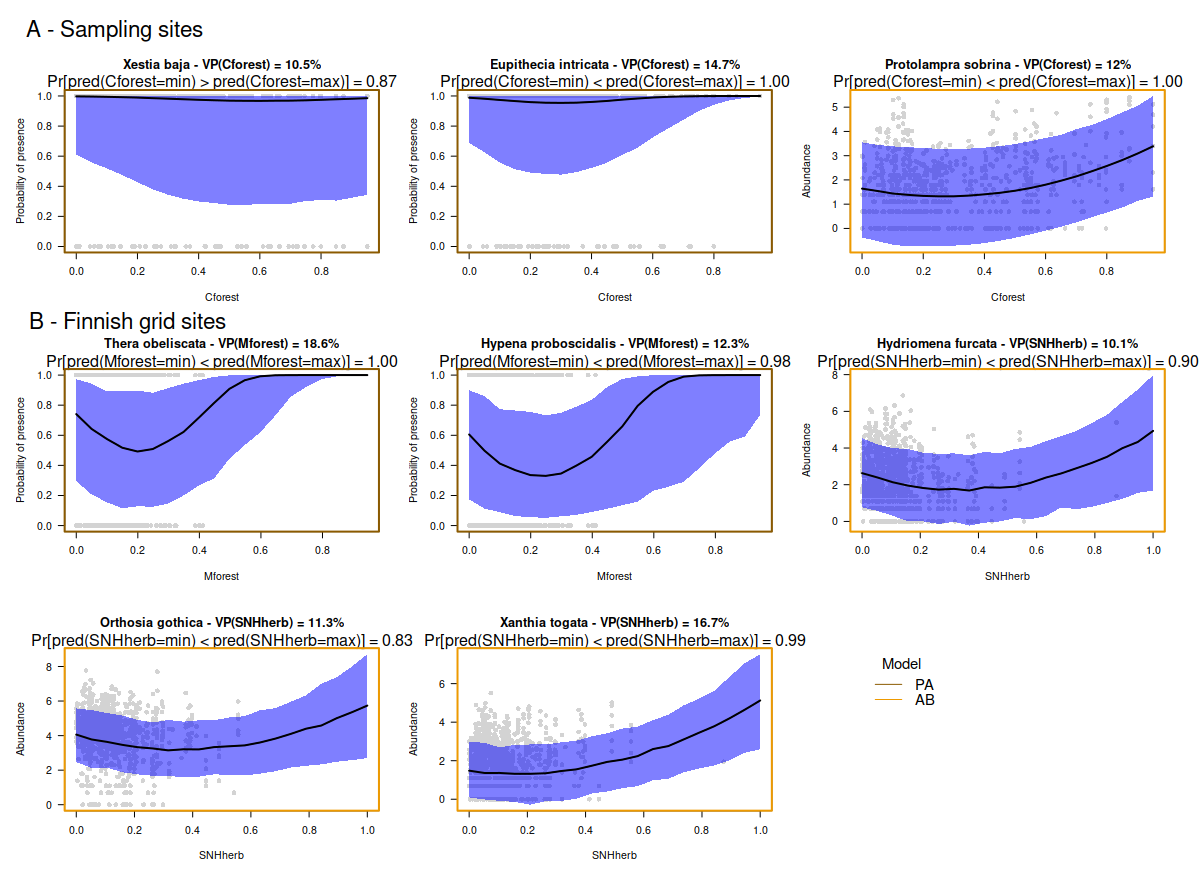


### Figure S1.7: Species response curves with ‘ushape’ like pattern at the sampling sites (A) and at the Finnish grid sites (B).

# Appendix S2: Supplementary results

## Model performances

### Table S2.1: Model fit statistics for the hurdle models with habitat delays

*To assess model fit, we display the potential scale reduction factors for the beta and gamma-parameters as well as the measures of performances (AUC and TjurR^2^ for the PA models and R^2^ for the abundance conditioned on presence model).*

*
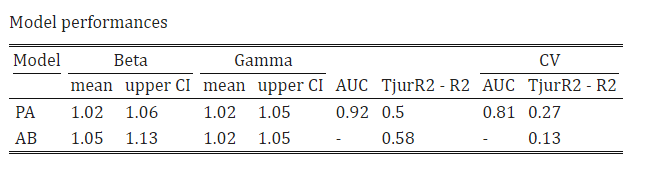
*

## Variance partitioning of moth occurrence and abundance – Sampling sites

At the level of sampling sites, the variance partitioning into the relative contributions of habitat and climate showed mostly similar patterns between data types (presence-absence, PA, and abundance conditioned on presence, AB; Fig. S2.1. The numbers are discussed in the main text and compared to the Finnish grid sites level.


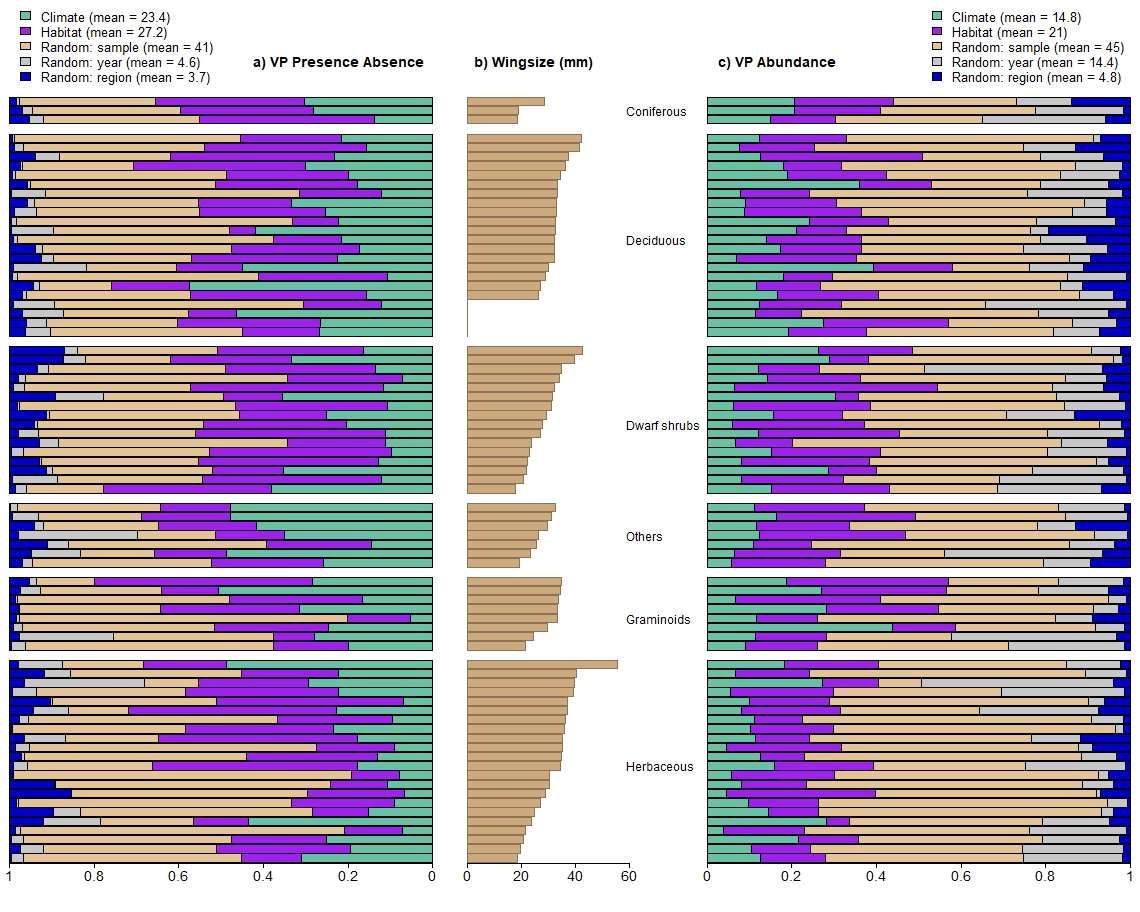


### Figure S2.1: Variance partitioning among grouped covariates (habitat vs. climate) across the sampling sites.

*The proportion of variation in species presence (a) and abundance conditioned on presence (c) as explained by environmental covariates as fixed effects and spatial and temporal covariates as random effects across the sampling sites. One row represents one species, and species are grouped by host plant form (as shown between b and c). Within each hostplant group, individual species are sorted by increasing wing span (from bottom to top - b).* *The rows order matches the order in table S1.1.*

Out of the 78 focal species and across the sampling sites, habitat was the dominated driver of occurrence for 46 ([40; 53]) species and of abundance for 52 ([45; 59]) species.

For heterogeneous sites, variability in presence-absence was driven equally by variability in climatic and habitat conditions. At sites dominated by water and forest, we found slightly more variability attributed to climatic than habitat conditions. In forest-dominated sites, variability in habitat variables explained more variation in moth occurrences than it did at water-dominated sites. Wetland and vegetated sites were the only sites where variability in occurrences was mostly explained by habitat conditions (Fig. S2.2).

To assess the strength of the context-dependence of the relative effects of climate and habitat on moth community, we also calculated the between and within group variance partitions. Both PA and AB models showed comparable average variance within and between groups for habitat covariates. Only for the PA data model did we observe larger within group variation for climatic covariates.


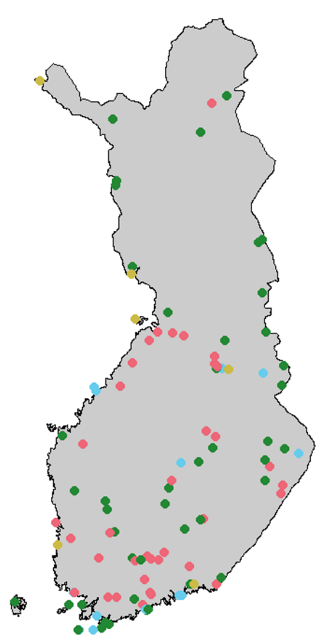

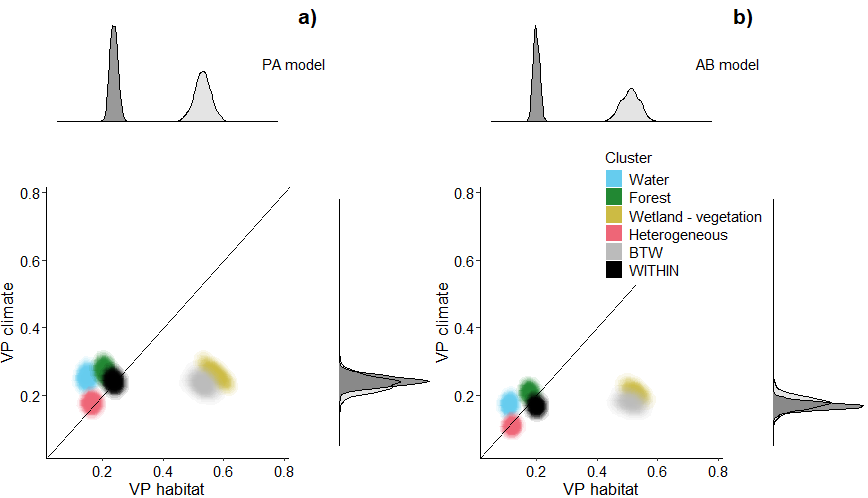


### Figure S2.2: Context-dependence of the importance of habitat vs. climate for moth occurrence (PA) and abundance (AB) at sampling sites.

*The map on the left shows the classification of sampled sites into four main habitat profiles using k-means clustering. The heat plots in the panels a) and b) show the joint posterior distributions of the proportion of variance explained by habitat (x-axis) and climate (y-axis) across locations within each of the four main habitat profiles (heterogeneous, forest, wetland, and water; red to blue colours) and the partition of the total variance into variation WITHIN and between (BTW) habitat profiles (red and orange colors). The marginal posterior distributions of the within-between partition are shown by the density plots along the margins of the figures. For patterns at the level of predictive sites across Finland, see Fig. 2 of the main text.*

Compared to patterns at the level of the Finnish grid scale presented in the main text, we noted a few differences at the level of sampling sites (Fig. S2.3). Overall, habitat showed less explanatory power, matching the results shown in Fig. S2.1 and Fig. S2.2. Compared to the results across Finnish grid cells, species grouped by wing-span displayed similar patterns at the sites levels.


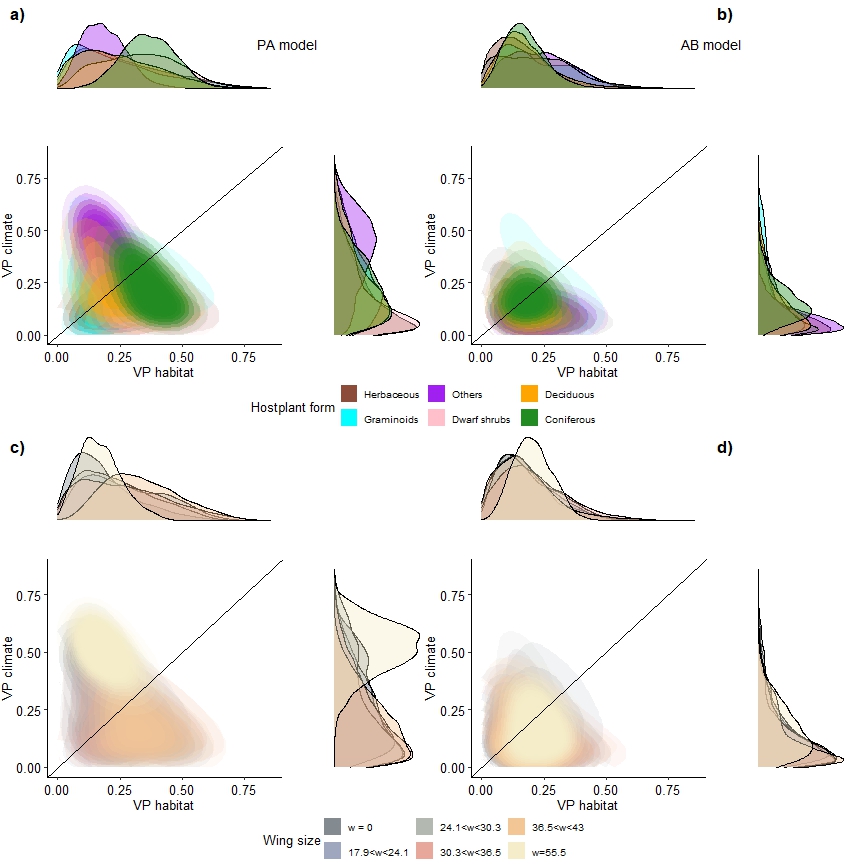


### Figure S2.3: Variation in the importance of habitat vs. climate for the occurrence (PA (presence/absence) model – panels a and c) and abundance (AB model –panels c and d) of moths associated with different traits at the sampling sites.

*The top row (panels a and b) shows results for species grouped by host plant growth form, whereas the bottom row shows results for species grouped by wing span (panels c and d). Each subplot summarizes the joint posterior distributions (heatmap) of the proportion of variance explained by habitat (x-axis) vs. climate (y-axis) among species sharing the same growth form of their host plants (graminoids, herbaceous plants, dwarf shrubs, coniferous trees, deciduous trees and other* *food sources taxa) or sharing similar wing span (W=0mm, 17.9<W<24.1mm, 24.1mm<W<30.3mm, 30.3mm<W<36.5mm, 36.5mm<W<43mm, and W=55.5mm.).*

## Environmental changes within sampling sites and across Finland

Supplementary Figures S2.4 and S2.5 describe the average changes in environmental conditions over the study period. All habitat and climate covariates have experienced clear changes across Finland over the study period of 1999-2020 (Supplementary Figures S2.4 and S2.5). Apart from precipitation, average climatic conditions displayed a clear latitudinal gradient (Supplementary Figure S2.4). Overall, the number of chilling degree-days has decreased over time, whereas growing degree-days have generally increased during the study period (Supplementary Figure S2.4f, and g). Snow depth has increased in the north but decreased in the south of Finland (Supplementary Figure S2.4h). Precipitation changes does not follow any clear pattern (Supplementary Figure S2.4e). The variability of the summer growing degree days and snow depth is larger in places where the trend was the steepest (Supplementary Figure S2.4k and l). For precipitation and winter chilling degree-days, we noticed no corresponding change in variability (Supplementary Figure S2.4i and j).

Coniferous forests and mixed forests dominate the overall Finnish landscape, whereas broad-leaved forests and semi-natural herbaceous habitats are predominant in the northern part of the country (Supplementary Figure S2.5a, b, c and d). Across Finland, forest patches are largely aggregated, and the landscape is diverse (Supplementary Figure S2.5e, and f). Apart from the most northern part and a few sites in the southern east, the proportion of conifers have increased over time, while the proportion of broad-leaved forest, mixed forest and semi natural habitats have decreased (Supplementary Figure S2.5g, h, i and j). Landscape diversity has also decreased, similarly to the aggregation of forest patches over time (Supplementary Figure S2.5k, and l). The highest variability in estimated trends occurred in the proportion of mixed forest and in landscape diversity (Supplementary Figure S2.5o, and r). Other habitat covariates showed less variability (Supplementary Figure 5m, n, p, and q).

Importantly, the distributions of habitat and climate changes occurring over the sampling sites did not suffice to characterize the changes evident across the full grid of sites distributed across Finland (Supplementary Figure S2.4 and S2.5; violin plots below panels).

***
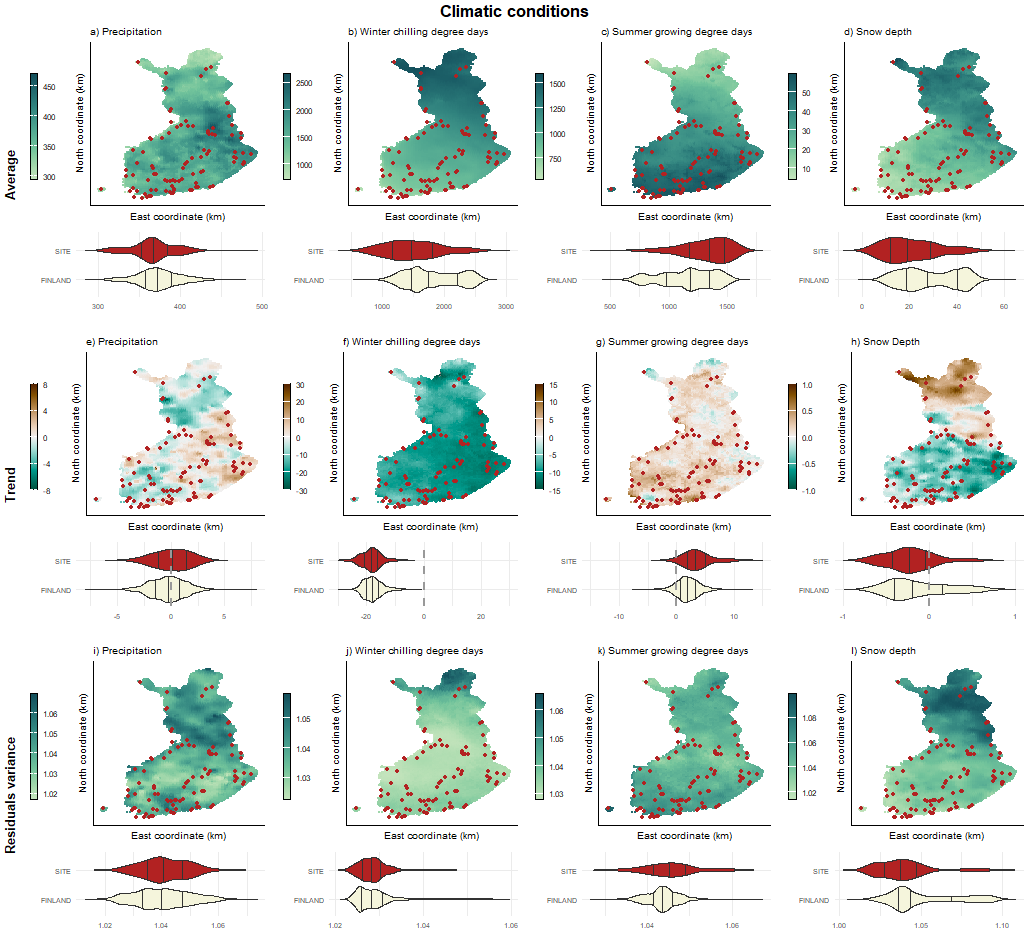
***

Figure S2.4: Summary of climatic changes in Finland*.*

*Each row of panels represents a different aspect of change: 1. The average value over the years, 2. The local (point-wise) trend estimated from the linear model and 3. The variability in standardized residuals (variance). To summarize information at the levels of all of Finland vs. the monitored sites, each map is combined with two violin plots (in beige and red, respectively). Each column of panels shows changes in a.e.i: precipitation, b.f.j. winter chilling degree-days, c.g.k. summer growing degree-days, d.h.l. snow depth.*

*
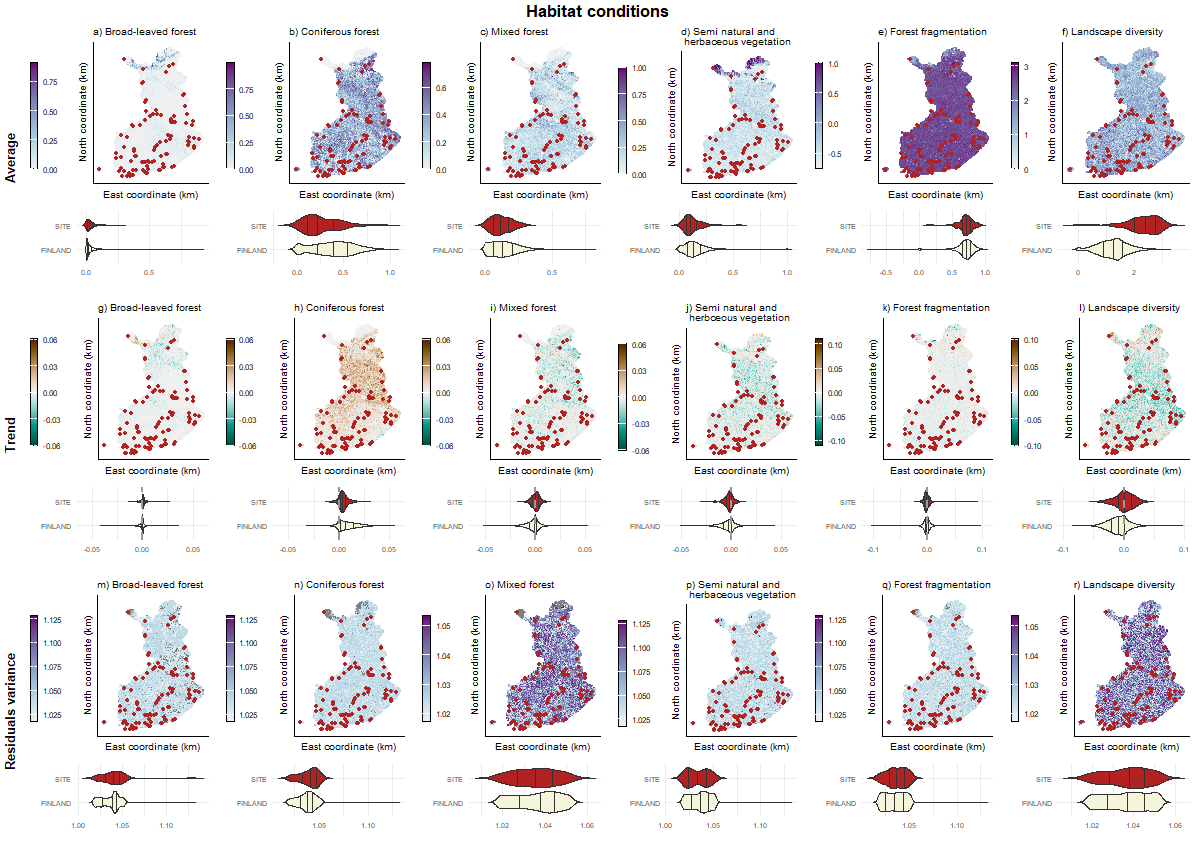
*

Figure S2.5: Summary of habitat changes in Finland.

*Each row of panels represents a different aspect of change: 1. The average value over the years, 2. the local (point-wise) trend estimated from the linear model and 3. the variability in standardized residuals (variance). To summarize information at the levels of all of Finland vs. the monitored sites, each map is combined with two violin plots (in beige and red, respectively). Each column of panels shows changes in a.g.m. proportion of broad-leaved forest, b.h.n. proportion of coniferous forest, c.i.o. proportion of mixed forest, d.j.p. proportion of semi natural herbaceous habitat, e.k.q. fragmentation and f.l.r. landscape diversity. Gray grid sites correspond to ‘NA’ as no values of habitat proportion or forest fragmentation could be measured.*

# Appendix S3: Code and data

Data available from the Dryad Digital Repository <https://doi.org/10.5061/dryad.0k6djhb5r> (Guilbault et al., 2025)

# References

Anderson, M.J., Walsh, D.C.I., Sweatman, W.L., Punnett, A.J., 2022. Non-linear models of species’ responses to environmental and spatial gradients. Ecol. Lett. 25, 2739–2752. https://doi.org/10.1111/ele.14121

Antão, L.H., Weigel, B., Strona, G., Hällfors, M., Kaarlejärvi, E., Dallas, T., Opedal, Ø.H., Heliölä, J., Henttonen, H., Huitu, O., Korpimäki, E., Kuussaari, M., Lehikoinen, A., Leinonen, R., Lindén, A., Merilä, P., Pietiäinen, H., Pöyry, J., Salemaa, M., Tonteri, T., Vuorio, K., Ovaskainen, O., Saastamoinen, M., Vanhatalo, J., Roslin, T., Laine, A.-L., 2022. Climate change reshuffles northern species within their niches. Nat. Clim. Change 12, 587–592. https://doi.org/10.1038/s41558-022-01381-x

Brock, G., Pihur, V., Datta, Susmita, Datta, Somnath, Brock, M.G., Biobase, S., 2021. Package ‘clValid.’

Esbjerg, P., Lauritzen, A., 2010. Oviposition response of the Turnip moth to soil moisture. Acta Agric. Scand. Sect. B-Soil Plant Sci. - ACTA AGR SCAND SECT B-SOIL PL 60, 89–94. https://doi.org/10.1080/09064710902741956

Fält-Nardmann, J.J.J., Ruohomäki, K., Tikkanen, O.-P., Neuvonen, S., 2018. Cold hardiness of Lymantria monacha and L. dispar (Lepidoptera: Erebidae) eggs to extreme winter temperatures: implications for predicting climate change impacts. Ecol. Entomol. 43, 422–430. https://doi.org/10.1111/een.12515

Guilbault, E., Sihvonen, P., Suuronen, A., Huikkonen, I.-M., Pöyry, J., Laine, A.-L., Roslin, T., Saastamoinen, M., Vanhatalo, J., 2025. Data from: Strong context-dependence in the relative importance of climate and habitat on macro-moth community changes in Finland. Data available from the Dryad Digital Repository. https://doi.org/10.5061/dryad.0k6djhb5r

Habel, J.C., Segerer, A.H., Ulrich, W., Schmitt, T., 2019. Succession matters: Community shifts in moths over three decades increases multifunctionality in intermediate successional stages. Sci. Rep. 9, 5586. https://doi.org/10.1038/s41598-019-41571-w

Huikkonen, I.-M., Korhonen, P., Leinonen, R., Suuronen, A., Pöyry, J., 2024. Valtakunnallinen yöperhosseuranta 30 vuotta (1993–2022) (No. 26), Suomen ympäristökeskuksen.

Jalas, I., 1960. Eine leichtgebaute, leichttransportable Lichtreuse zum Fangen von Schmetterlingen. Ann Entomol Fenn 26, 44–50.

Jansen, F., Oksanen, J., 2013. How to model species responses along ecological gradients – Huisman–Olff–Fresco models revisited. J. Veg. Sci. 24, 1108–1117. https://doi.org/10.1111/jvs.12050

Lintott, P.R., Bunnefeld, N., Fuentes-Montemayor, E., Minderman, J., Blackmore, L.M., Goulson, D., Park, K.J., 2014. Moth species richness, abundance and diversity in fragmented urban woodlands: implications for conservation and management strategies. Biodivers. Conserv. 23, 2875–2901. https://doi.org/10.1007/s10531-014-0753-z

Luoto, M., Heikkinen, R.K., Pöyry, J., Saarinen, K., 2006. Determinants of the biogeographical distribution of butterflies in boreal regions. J. Biogeogr. 33, 1764–1778. https://doi.org/10.1111/j.1365-2699.2005.01395.x

Merckx, T., Dantas de Miranda, M., Pereira, H.M., 2019. Habitat amount, not patch size and isolation, drives species richness of macro-moth communities in countryside landscapes. J. Biogeogr. 46, 956–967. https://doi.org/10.1111/jbi.13544

Merckx, T., Feber, R.E., Hoare, D.J., Parsons, M.S., Kelly, C.J., Bourn, N.A.D., Macdonald, D.W., 2012. Conserving threatened Lepidoptera: Towards an effective woodland management policy in landscapes under intense human land-use. Biol. Conserv. 149, 32–39. https://doi.org/10.1016/j.biocon.2012.02.005

Neuvonen, S., Virtanen, T., 2015. Abiotic factors, climatic variability and forest insect pests. Clim. Change Insect Pests CAB Int. Wallingford 154–172.

Niittynen, P., Luoto, M., 2018. The importance of snow in species distribution models of arctic vegetation. Ecography 41, 1024–1037. https://doi.org/10.1111/ecog.03348

Öckinger, E., Schweiger, O., Crist, T.O., Debinski, D.M., Krauss, J., Kuussaari, M., Petersen, J.D., Pöyry, J., Settele, J., Summerville, K.S., Bommarco, R., 2010. Life-history traits predict species responses to habitat area and isolation: a cross-continental synthesis. Ecol. Lett. 13, 969–979. https://doi.org/10.1111/j.1461-0248.2010.01487.x

Ovaskainen, O., Abrego, N., 2020. Joint species distribution modelling: With applications in R. Cambridge University Press.

Ovaskainen, O., Tikhonov, G., Norberg, A., Guillaume Blanchet, F., Duan, L., Dunson, D., Roslin, T., Abrego, N., 2017. How to make more out of community data? A conceptual framework and its implementation as models and software. Ecol. Lett. 20, 561–576.

Pöyry, J., Böttcher, K., Fronzek, S., Gobron, N., Leinonen, R., Metsämäki, S., Virkkala, R., 2018. Predictive power of remote sensing versus temperature‐derived variables in modelling phenology of herbivorous insects. Remote Sens. Ecol. Conserv. 4, 113–126.

Pöyry, J., Leinonen, R., Söderman, G., Nieminen, M., Heikkinen, R.K., Carter, T.R., 2011. Climate-induced increase of moth multivoltinism in boreal regions. Glob. Ecol. Biogeogr. 20, 289–298. https://doi.org/10.1111/j.1466-8238.2010.00597.x

Rissanen, T., Niittynen, P., Soininen, J., Luoto, M., 2021. Snow information is required in subcontinental scale predictions of mountain plant distributions. Glob. Ecol. Biogeogr. 30, 1502–1513. https://doi.org/10.1111/geb.13315

Schulz, T., Saastamoinen, M., Vanhatalo, J., 2025. Model-based variance partitioning for statistical ecology. Ecol. Monogr. 95, e1646. https://doi.org/10.1002/ecm.1646

Slade, E.M., Merckx, T., Riutta, T., Bebber, D.P., Redhead, D., Riordan, P., Macdonald, D.W., 2013. Life‐history traits and landscape characteristics predict macro‐moth responses to forest fragmentation. Ecology 94, 1519–1530.

Summerville, K.S., Crist, T.O., 2004. Contrasting effects of habitat quantity and quality on moth communities in fragmented landscapes. Ecography 27, 3–12. https://doi.org/10.1111/j.0906-7590.2004.03664.x

Tikhonov, G., Duan, L., Abrego, N., Newell, G., White, M., Dunson, D., Ovaskainen, O., 2020. Computationally efficient joint species distribution modeling of big spatial data. Ecology 101, e02929. https://doi.org/10.1002/ecy.2929

Uhl, B., Wölfling, M., Bässler, C., 2022. Mediterranean moth diversity is sensitive to increasing temperatures and drought under climate change. Sci. Rep. 12, 14473. https://doi.org/10.1038/s41598-022-18770-z

Virtanen, T., Neuvonen, S., 1999. Performance of moth larvae on birch in relation to altitude, climate, host quality and parasitoids. Oecologia 120, 92–101.
